# Designing a next generation solar crystallizer for real seawater brine treatment with zero liquid discharge

**DOI:** 10.1038/s41467-021-21124-4

**Published:** 2021-02-12

**Authors:** Chenlin Zhang, Yusuf Shi, Le Shi, Hongxia Li, Renyuan Li, Seunghyun Hong, Sifei Zhuo, Tiejun Zhang, Peng Wang

**Affiliations:** 1 Water Desalination and Reuse Center, Division of Biological and Environmental Science and Engineering, King Abdullah University of Science and Technology, Thuwal, 23955-6900 Saudi Arabia; 2 Department of Mechanical Engineering, Masdar Institute, Khalifa University of Science and Technology, PO Box 127788, Abu Dhabi, United Arab Emirates; 3 Department of Civil and Environmental Engineering, The Hong Kong Polytechnic University, Hong Kong, China

**Keywords:** Solar thermal energy, Civil engineering

## Abstract

Proper disposal of industrial brine has been a critical environmental challenge. Zero liquid discharge (ZLD) brine treatment holds great promise to the brine disposal, but its application is limited by the intensive energy consumption of its crystallization process. Here we propose a new strategy that employs an advanced solar crystallizer coupled with a salt crystallization inhibitor to eliminate highly concentrated waste brine. The rationally designed solar crystallizer exhibited a high water evaporation rate of 2.42 kg m^−2^ h^−1^ under one sun illumination when treating real concentrated seawater reverse osmosis (SWRO) brine (21.6 wt%). The solar crystallizer array showed an even higher water evaporation rate of 48.0 kg m^−2^ per day in the outdoor field test, suggesting a great potential for practical application. The solar crystallizer design and the salt crystallization inhibition strategy proposed and confirmed in this work provide a low-cost and sustainable solution for industrial brine disposal with ZLD.

## Abstract (web summary)

Proper disposal of industrial brine remains a critical environmental challenge. Here, the authors devise a solar crystallizer and propose a salt crystallization inhibition strategy, which together provide a low-cost and sustainable solution for industrial brine disposal with zero liquid discharge.

## Introduction

The modern industries (e.g., seawater desalination, mining, petrochemical, food, and power generation) produce concentrated waste brine byproducts every day^1–6^. The volumes of these brines range from hundreds of liters to tens of thousands of cubic meters per day, depending on the type and scale of the industrial sectors. Currently, most of the waste brines, especially brines produced in seawater desalination plants, are directly discharged into adjacent open water bodies, e.g., rivers, lakes, near shore seawater, and the rest are injected underground in deep wells or treated in evaporation ponds^7–10^. These conventional methods have been proved to have detrimental effects on aquatic ecosystems and land vegetation systems^11–14^. Thus, with ever tightening environmental regulations and increasing public environmental awareness, these direct or semi-direct disposal of the waste brines is facing strong criticism. As an ambitious target for brine treatment, the concept of zero liquid discharge (ZLD) has received renewed attentions recently, since it aims to eliminate all waste liquid and produce solid salts as the only byproduct^7,11,15^.

A typical ZLD process is composed of a concentration system, which concentrates the original source brine to near saturation, and a crystallization system that completely removes all residual water from the brine to produce solid salts^11^. In the past decades, the concentration system has been significantly improved by various technologies, including reverse osmosis (RO), electrodialysis, membrane distillation (MD), and mechanical vapor compression concentrator, etc. However, the improvement in the crystallization system is very sluggish because all membrane based desalination technologies cannot work with near-saturation brines due to salt scaling and fouling^7,11^. The current brine crystallization is mainly achieved either by brine crystallizers or by evaporation ponds^16^. The conventional brine crystallizers use electricity or fossil fuel to heat the brines for bulk water evaporation and thus salt crystallization with high energy consumption (>50 kWh m^−3^)^12,16,17^. In addition, the use of pressure container made from expensive corrosion resistant materials brings high capital cost of the brine crystallizers^12,16^. The application of evaporation pond, on the other hand, is limited by its restricted capacity, high land cost, and low solar energy efficiency^18,19^. Therefore, the crystallization system has been the bottleneck of the ZLD brine treatment, but it receives very little attention mainly because of the lack of new strategies.

Photothermal material assisted solar driven water evaporation is gaining popularity as an environmental friendly way to produce water vapor for clean water production via solar distillation^20–33^. In such a process, solar energy is harvested and converted to heat by the photothermal materials, and thus to produce water vapor from various source water, such as seawater, in a solar still. Then the condensate from the water vapor is collected as fresh water. Salt precipitation on the photothermal material during operation is considered troublesome because it affects the light absorption of the photothermal material^34^. Several rationally designed structures have been reported in the last several years to eliminate the surface salt precipitation by making salt rejecting photothermal devices via facilitating the accumulated salts diffusion back to the source water^27,35,36^.

In 2016, Mi et al.^37^ regarded the salt accumulation on the photothermal material as an opportunity to achieve ZLD desalination in a solar still setup. When 15 wt% pure NaCl aqueous brine was used as source water, a thick layer of accumulated NaCl crystals formed on top of their two dimensional (2D) graphene oxide (GO) membrane. The white salt layer significantly decreased light absorption of the device, and led to a considerable reduction in evaporation rate. However, a stable water evaporation rate of 0.5 kg m^−2^ h^−1^ was still recorded, indicating the salt precipitation did not block the evaporation. In 2018, a three dimensional (3D) cup-shaped solar evaporator which separated the light adsorption surface from the salt precipitation surface was reported. This design enabled an unimpeded sunlight absorption and in turn a much higher and long-term stable water evaporation rate (e.g., 1.26 kg m^−2^ h^−1^) even at a near saturated brine (25 wt% NaCl brine)^38^. It should be noted the above-mentioned evaporation rates were both achieved in open space without collecting the condensate. Even for the 3D cup structure, the clean water production rate was only ~0.5 kg m^−2^ h^−1^ when placed inside a solar still. Therefore, the direct ZLD desalination can be achieved by this advanced solar distillation but only with less than 35% energy efficiency for seawater.

One of the major advantages of solar driven water evaporation process is that the water removal rate is only slightly affected by the salt concentration of the source water, and latent heat of highly concentrated NaCl brine is lower than that of pure water^38,39^. On the contrary, the energy consumption and operation cost significantly increases with the increase of salt concentration in membrane-based process, especially RO^40^. Thus, instead of one process achieving all, it is a more attractive and energy efficient strategy to first use conventional processes (e.g., RO and MD) to produce clean water from regular seawater and to concentrate brines with low concentrations to near saturation, and then to employ the solar driven water evaporation for salts crystallization from the near-saturation brines to achieve ZLD goal.

**Fig. 1:**
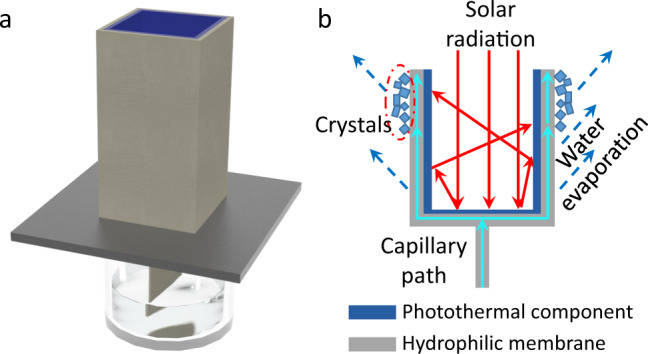
Schematic illustration of solar crystallization device. **a** A scheme of the solar crystallizer; **b** schematic of cross section view of the solar crystallizer.

In this work, we rationally designed a new 3D solar crystallizer device operated with dead-end type solar driven water removal mode (Fig. 1), in which water evaporation surface and light absorption surface are physically separated by an aluminum sheet with high thermal conductivity. Its bottom and inner wall act as the sunlight absorbing component with a high light absorptance of 0.99, while its outer wall surface serves as the water evaporation surface and consequently salt crystallization surface. The high thermal conductivity of the aluminum separator benefits effectively conducting the heat generated at the bottom of the device to its wall for water evaporation. All these features result in a high solar-to-vapor performance of the device. This device produced high water removal performance (1.61 kg m^−2^ h^−1^) under one sun illumination when treating pure NaCl brine with 24 wt% concentration. However, when directly treating concentrated real seawater brines, a quick decline followed by a close to zero water evaporation rate was recorded by the same solar crystallizer. Comparing the crystallization behavior of pure NaCl brine and real seawater reverse osmosis (SWRO) brine, it was found that the scaling formed by magnesium species, which has not received enough attention in current researches in this field, clogged water transport channels and then caused performance degradation. Here, the salt crystallization inhibitor, nitrilotriacetic acid (NTA), was introduced into the same real seawater source brines to modulate salt crystallization behaviors on the outer surface of the solar crystallizer, successfully, resulting in dense scaling crust layer-free salt crystallization while treating real brine. The application of the solar crystallizer coupled with small amount (only 8.4 wt‰ of the salt) of salt crystallization inhibitor was demonstrated for treating highly concentrated SWRO brine (21.6 wt%) for 288 h and showed a very high and stable water evaporation rate of 2.42 kg m^−2^ h^−1^. Moreover, the solar crystallizer array exhibited a daily water evaporation rate of 48.0 kg m^−2^ in the outdoor field test, showing a great potential for practical application. It is believed that this simple but promising strategy provides a low-cost and sustainable solution especially for small to medium-sized industrial brine treatment with ZLD, where both solar irradiation and access to land are available.

## Results

### Structure and design of the 3D solar crystallizer

Figure 1a presents a schematic diagram of the 3D solar crystallizer with an open box structure with one bottom closed. The bottom and wall of the crystallizer are bi-layered in configuration. The inner layer is a commercially available spectrally selective solar absorber (Alanod^®^) homogeneously coated on an aluminum sheet and serves as photothermal component. The outer layer of the solar crystallizer is a porous and hydrophilic quartz glass fibrous filter membrane (QGF membrane, Merck^®^) (Fig. S1). The outer QGF membrane, when wet, is directly stuck onto the backside of the aluminum sheet via capillary force without any glue. It wicks brine from the source brine reservoir and allows the brine to spread over the entire outer surface during operation. The inner layer of the 3D crystallizer acts as the light absorbing surface while the outer QGF membrane serves as water transportation and evaporation surface. The aluminum sheet completely separates the two active surfaces and has a desirably high thermal conductivity (~200 W m^−1^ K^−1^), which is beneficial for the heat conduction. The solar crystallizer is directly placed on top of an expanded polystyrene foam with a low thermal conductivity (*K* = 0.03 W m^−1^ K^−1^) to minimize the heat loss to the bulk brine^32^. The source brine is transported from the reservoir to the solar crystallizer by a one-dimensional (1D) QGF strip in the middle of the device via capillary action^41^.

When sunlight is illuminated onto the crystallizer directly from the above, it is absorbed by the photothermal coating at the bottom of the device to generate heat. As illustrated in Fig. 1b, the generated heat is then effectively conducted to the wall part owing to the high thermal conductivity of the aluminum sheet, and thereafter drives the water evaporation and the ultimately precipitation of salts exclusively on the outer wall of the crystallizer. By design, our 3D solar crystallizer completely physically separates its light absorbing surface and water evaporation and thus salt precipitation surface, which solves the drawback of precipitating salt crystals affecting light absorption otherwise inherent in 2D devices and allows for two surfaces to be independently optimized.

**Fig. 2:**
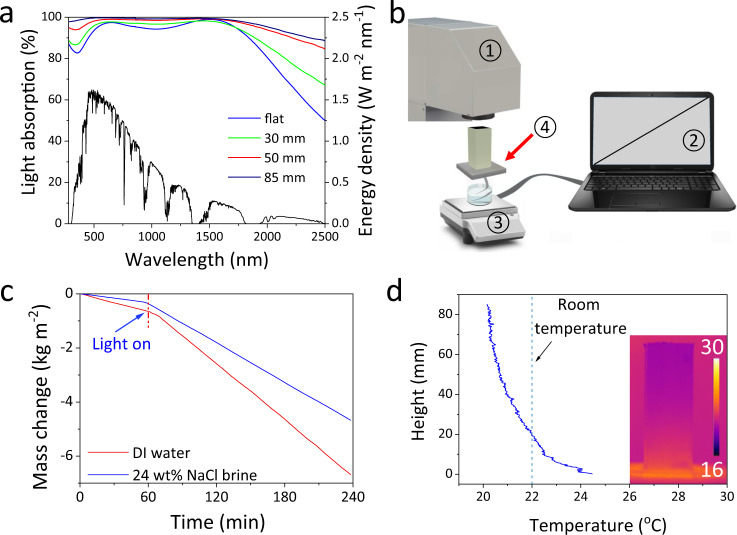
Evaporation performance of solar crystallizer. **a** The absorption spectra of the 3D solar crystallizers with different wall heights across 250–2500 nm, and standard AM 1.5 G solar spectrum; **b** the scheme of the labmade setup for solar evaporation performance measurement (1. Solar simulator, 2. computer, 3. electrical balance, 4. solar crystallizer); **c** the mass change curves of pure water and 24 wt% NaCl by the solar crystallizer under one sun illumination; **d** the outer surface temperature profile along the wall height of the 3D solar crystallizer in wet state under one sun illumination.

Considering the size and shape of the output beam of the available solar simulator (Newport 94021A), the designed 3D solar crystallizer was fabricated to have a tetragonal cup-shaped structure with the bottom side length of 31 mm. The inner surface of the wall is capable of recycling the diffuse reflection light from the bottom, and thus strengthens the light absorption of the device^30,41^. The 3D crystallizers with a wall height of 30, 50, and 85 mm have a solar absorptance of 0.96, 0.98, and 0.99, respectively (Fig. 2a, calculation details in Supplementary Note S1), which compares favorably against 0.94 of a flat photothermal 2D sheet with the same composition.

### Solar-driven water evaporation performance of pure water

The solar-driven water evaporation performance was evaluated by using a labmade setup under one-sun illumination as illustrated in Fig. 2b. The weight change of the device was recorded real time (Fig. 2c) which was then used to calculate water evaporation rate. The average evaporation rate of pure water under one sun illumination on this 3D crystallizer reached 2.09 kg m^−2^ h^−1^ with an apparent solar evaporation efficiency of 138.5% and a net solar evaporation efficiency of 94.3% (calculation details in Supplementary Note S1). The efficient utilization of solar energy can be attributed to that the wall of the cup structure can efficiently recover the energy loss from the bottom light absorbing surface as discussed in literature^41^. The high apparent efficiency can be explained by that the device can gain additional heat from the surroundings, because the majority of the solar crystallizer is cooler than its surroundings even under sunlight (Fig. 2d)^30,42–44^. These results demonstrate that this 3D solar crystallizer produces a state-of-the-art performance in solar-driven water evaporation.

When the height of the solar crystallizer was further increased to 115 and 145 mm, the corresponding evaporation rates under one sun illumination increased to 2.39 kg m^−2^ h^−1^ and 2.70 kg m^−2^ h^−1^, respectively. Here, a COMSOL model (Supplementary Note S2) was established to gain insight into the relationship between the performance and height of the solar crystallizer. The modeling results reveal that, once the solar crystallizer’s height is over a certain value, the average temperature of the evaporation surface of the crystallizer is lower than the ambience, which forces environmental thermal energy flowing into the solar crystallizer. It is further shown that the evaporation rate well above the theoretical limit of a full solar-energy utilization is achieved therein and it increases almost linearly with the height of the solar crystallizer, which is in a good agreement with the experimental results measured in lab (Fig. S2). However, it is advised that the trade-off between the performance and material cost of the system should be carefully balanced in the practical applications. In this work, the wall height of the crystallizers was fixed at 85 mm thereafter.

### Solar crystallization of pure NaCl brines

**Fig. 3:**
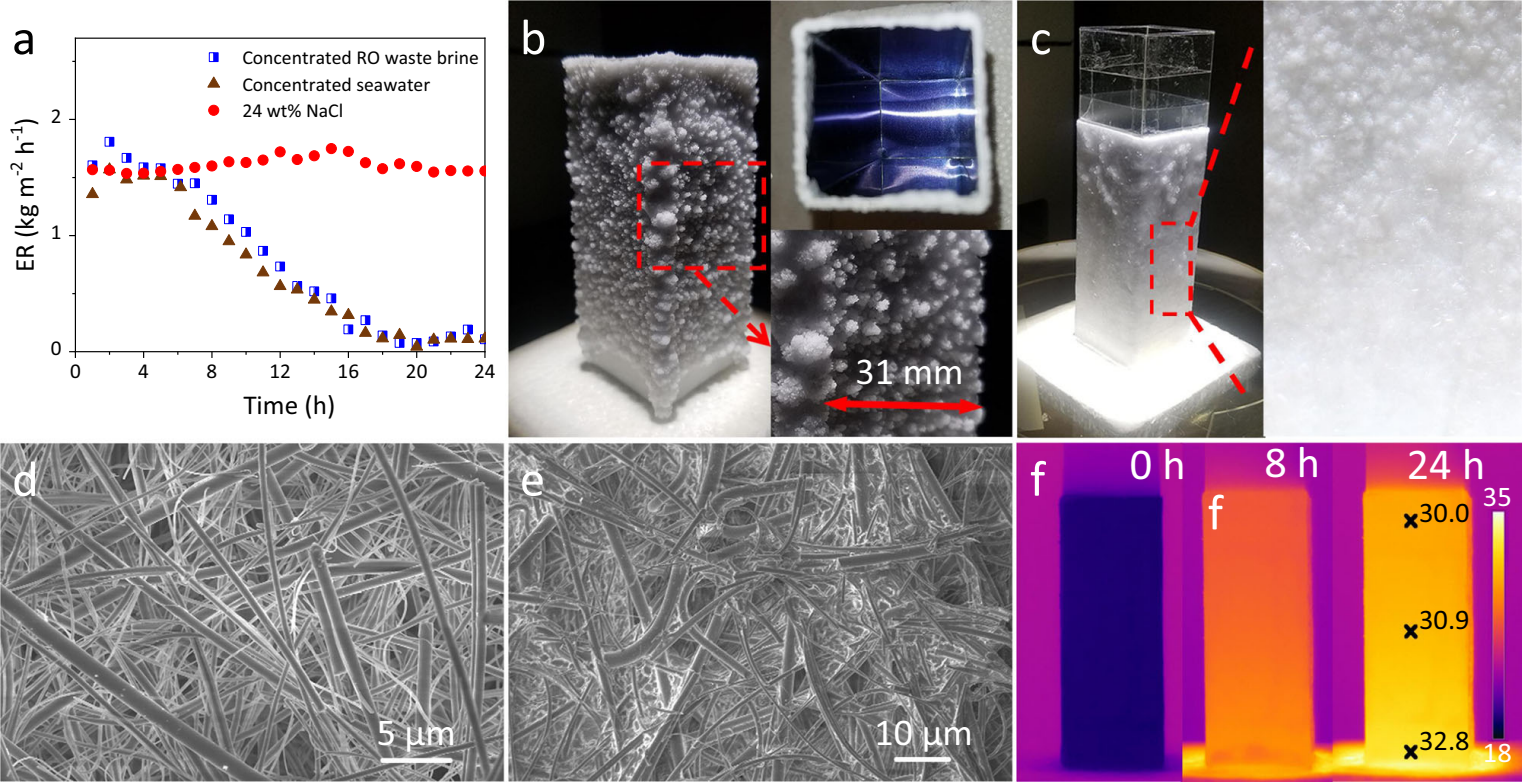
Solar evaporation and crystallization behavior of brines. **a** The evaporation rate (ER) of 24 wt% NaCl brine and real seawater brines in the 24-h operation; photos of the solar crystallizers after the 24-h operation using **b** 24 wt% NaCl brine and **c** concentrated RO waste brine; SEM images of the inner side of QGF membrane after operating with **d** 24 wt% NaCl brine and **e** concentrated real RO waste brine; **f** the IR images of the solar crystallizer while treating concentrated RO waste brine. Note: In Fig. 3c, an optional transparent polycarbonate shield with a height of 30 mm was added to the solar crystallizer in order to prevent salt crystal from creeping into the inner side of the crystallizer.

When the 3D solar crystallizer was operated with a 24 wt% pure NaCl brine, a stable high evaporation rate of 1.61 kg m^−2^ h^−1^ was recorded (Fig. 2c), which stayed steady for at least 24 h (Fig. 3a). This small decreases (~23%) in the evaporation rate of the NaCl brines relative to pure water is ascribed to the decrease in saturation vapor pressure due to the colligative property of brine, directly pointing to the clear advantage of thermal-based versus membrane-based process in treating highly concentrated brines^38^. After 24 h, the significant amount of salt crystals precipitated on the entire outer wall surface, forming a thick crust layer (Fig. 3b). The crust layer was composed of rough NaCl crystal balls with diameters in the range of 1.8–8.3 mm and the NaCl crystal balls were loosely packed together with plentiful void space among them. As a matter of fact, the crust layer of NaCl crystal balls could be removed by a stainless steel scraper (Fig. S3 and Supplementary video).

The SEM image of the inner side, the side that directly contacted the aluminum sheet, of the QGF membrane after 24 h of operation with 24 wt% NaCl brine is presented in Fig. 3d. It clearly shows that the inner side of the QGF membrane was quite clean with negligible salt crystals observed. The clean inner side and the thick salt crust layer on the outer surface can be explained as follows.

In the 3D crystallizer, heat is conducted from the aluminum sheet surface to the inner side first and then further to the outer surface of the QGF membrane. In addition, water evaporation is endothermic and an interfacial process, which takes place only at the outer surface of the QGF membrane. As a result, the outer surface of the QGF membrane possesses a lower temperature and higher salt concentration than the inner side of the same membrane, leading to a selective precipitation of NaCl salt crystals only on the outer surface^38^. The competitive advantage of the outer surface in salt precipitation keeps the inner side of the membrane free of salt crystals (Fig. 3d) and maintains the water channel inside the QGF membrane unobstructed.

The stable solar energy input, highly porous structure of the salt crust layer and the unobstructed water channels inside the QGF membrane all contribute to the stable water evaporation rate of the 3D solar crystallizer for treating even very highly concentrated NaCl brines. In conventional 2D solar crystallizers, owing to the coincidence of their light absorption and salt precipitation surfaces, they can only produce a small water evaporation rate in treating NaCl brines (e.g., 0.5 kg m^−2^ h^−1^ for 15 wt% NaCl brine), because the light absorption is significantly affected by the salt crust layer formed on their surfaces^37^. However, the evaporation rate, although small, can stay stable due to the highly porous NaCl salt crust layer that permits water transport, with which the results of this work agree well.

It is worth noting the 3D solar crystallizer in this work exhibited a significantly higher water evaporation rate (i.e., 1.61 kg m^−2^ h^−1^ even for 24 wt% NaCl brine) than the 3D solar device reported by Shi et al.^38^ with similar 3D cup-shaped structure (i.e., 1.26 kg m^−2^ h^−1^ for 25 wt% NaCl brine). The higher heat conductivity of the 3D crystallizer in this work may be the major reason. In these two 3D structures, the light directly hits only on the bottom part of the devices and is in situ converted to heat via photothermal effect. The low thermal conductivity of the device reported by Shi et al. makes its bottom part having a much higher temperature than the wall part, which increases the heat loss via thermal radiation. As a proof, our 3D crystallizer showed a relatively uniform temperature distribution under solar radiation (Fig. S4). We believe the uniform temperature profile across the wall in our device leads to its better performance.

### Solar crystallization of real seawater brines

The concentrated seawater with total salt concentration of 16.8 wt% and concentrated SWRO brine with total salt concentration of 21.6 wt% were prepared from concentrating Red Sea seawater and waste brine from a seawater RO desalination plant, respectively. The samples preparation details can be found in the “Experimental” section. Beside sodium and chlorine, the other major ions in the real brines include magnesium, calcium, and sulfate (Table S1).

Under otherwise the same operation conditions, the 3D solar crystallizer exhibited a relatively stable water evaporation rates during the first 4 h: 1.49 and 1.65 kg m^−2^ h^−1^ for the concentrated seawater and concentrated SWRO brine, respectively (Fig. 3a). The result that the concentrated SWRO brine with a higher salt concentration (i.e., 21.6 wt%) showed a slightly higher water evaporation rate than the concentrated seawater (i.e., 16.8 wt%) is a surprise, which presumably is caused by their different compositions (Table S1).

However, after the first 4 h, a significant reduction in the water evaporation rates was observed for both real brines. The evaporation rates quickly plummeted to near 0.1 kg m^−2^ h^−1^ by the end of 20 h (Fig. 3a). Due to the almost zero water evaporation, it is considered that the 3D solar crystallizer had failed at that point. This evaporation rate decay behavior observed for both real seawater brines stands as a sharp contrast against the very stable evaporation behavior of the pure NaCl brines by the same 3D solar crystallizer, which is unexpected and has never been reported in the literature for advanced solar crystallizers.

To ascertain the reason behind the quite contrasting water evaporation behaviors, a 2D silica/carbon/silica tri-layered coaxial fibrous membrane disk (2D SCS disk)^38^ and a 3D graphene oxide/multiwalled carbon nanotube coated cellulose membrane film (3D GO/MWCNT film)^20^ were fabricated according to the literatures and used as solar crystallizers. The significant decrease in the water evaporation rates was also observed on these two devices within 2 h when treating the concentrated SWRO brine (Fig. S5a). In these cases, when the water evaporation rates had dropped to almost zero, these crystallizer surfaces appeared quite black, indicating the light absorption of these crystallizers was not significantly degraded by the precipitated salt layer (Fig. S5b, c). Thus, it is believed that the variation in the light absorption was not the real cause of the degraded water evaporation rates by the 3D crystallizer while treating the real brines.

After 24-h of operation with the concentrated SWRO brine, the outer wall surface of the 3D crystallizer in this work was covered with a dense and glass-like salts layer (Fig. 3c). The EDS analysis of the dense layer solids (Fig. S6) shows that they contained sodium, chlorine, magnesium, calcium, and sulfur elements. On the XRD pattern (Fig. S7a) of the wet salt crust layer, highly crystalized NaCl with rock salt phase structure and MgSO_4_·7H_2_O with orthorhombic epsomite phase were clearly observed. After drying at 40 °C, the MgSO_4_·7H_2_O phase was converted to MgSO_4_·6H_2_O according to the XRD pattern (Fig. S7b). No CaSO_4_·2H_2_O or CaSO_4_·0.5 H_2_O could be found in the XRD pattern, which may be due to the small amount of CaSO_4_. The SEM image unexpectedly disclosed that the inner side of the QGF membrane was completely filled with the solid salts after 24-h operation with the real concentrated SWRO brines (Fig. 3e), which is also confirmed by EDS analysis (Fig. S8). The porosity of QGF membrane (measured by mercury intrusion porosimetry) decreased from 68.20 to 1.00% after the formation of salt scaling. The clogged pores are unable to transport water from the source brine to the evaporation surface.

Moreover, as the evaporation rate was decaying with time, the temperature of the crystallizer gradually increased (Fig. 3f) as a way of releasing extra heat into the environment so to reach a new equilibrium with the stable and unaffected solar influx. The temperature of the crystallizer was as high as 32.8 °C, even higher than the dry solar crystallizer under one sun illumination (29.5 °C, Fig. S9).

The salt scaling leads to an almost complete loss of water evaporation capability by our 3D solar crystallizer for real brines. Therefore, until this problem is solved, the solar crystallizer would not perform to treat real seawater brines, although it shows an excellent performance in treating pure NaCl brines.

### Effect of salt crystallization inhibitor

Salt crystallization inhibitors are known to have the capability of effectively controlling the morphology of precipitating salts even at a very small amount. In this work, nitrilotriacetic acid (NTA), an effective and widely used salt crystallization inhibitor, was adopted due to its low cost and biodegradability^45,46^.

**Fig. 4:**
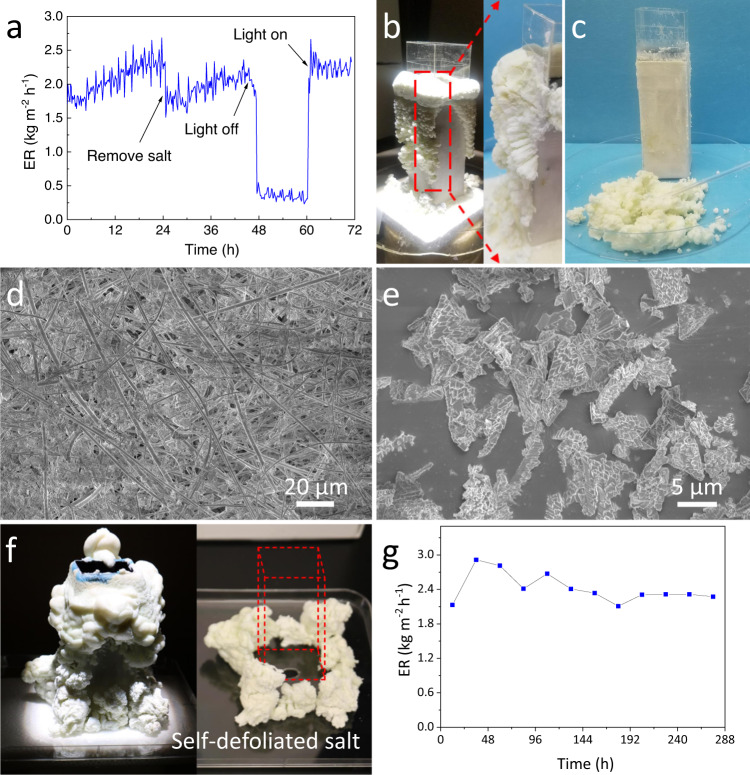
Solar evaporation and crystallization behavior of real seawater brine in the presence of NTA. **a** The water evaporation rate of the solar crystallizer using concentrated SWRO brine with 8.4‰ NTA; **b** photo image of the solar crystallizer and accumulated salt after 24-h operation; **c** photo image of the solar crystallizer after removing accumulated salt after 24-hour operation; SEM images of **d** QGF membrane after 24-h operation and **e** the salt crystals collected from the outer surface; **f** photo image of the solar crystallizer and self-defoliated salt after 48-h operation (the red dotted frame represents the position of removed solar crystallizer); **g** the water evaporation rate of the solar crystallizer during the 288-h operation (each point on the curve was the average evaporation rate of 24 h).

In using it, 8.4‰ NTA was added into the concentrated SWRO brine to investigate its effect (8.4‰ being the weight of NTA equal to 8.4‰ of that of the total salts in the brine). With the NTA in the brine, the average water evaporation rate of the concentrated SWRO brine by the 3D solar crystallizer in the first 24 h was lifted to 2.08 kg m^−2^ h^−1^, 22% higher than the water evaporation rate of 21.6 wt% pure NaCl brine (1.71 kg m^−2^ h^−1^, Fig. S10). The water evaporation rate stayed stable and even gradually and slightly increased during operation (Fig. 4a). The highest surface temperature of the 3D crystallizer was 28.4 °C (Fig. S11) under solar radiation, which was 4.4 °C lower than the case without NTA (Fig. 3f). The lower surface temperature is an indicator of the higher water evaporation performance of the crystallizer in the presence of NTA

After 24-h of operation, a layer of wet and fluffy salt crust layer was formed on the outer wall of the device with NTA in the concentrated SWRO brine (Fig. 4b), which is quite different from the dense glass-like salt crust layer formed in the absence of NTA. The presence of the accumulated salts did not decrease evaporation performance. On the contrary, the fluffy salt curst layer extended the actual water evaporation area (Fig. S12), which could explain the increase in water evaporation rate after adding NTA. The salt layer could be easily removed from the wall surface by a plastic spatula (Fig. 4c and Supplementary video) or by a mild shock impact (see Supplementary video). SEM observation results show that the salt precipitation did not plug the pores of the QGF membrane in this case (Fig. 4d).

After the salt crust layer was directly removed by a plastic spatula without any water washing treatment, the 3D solar crystallizer was able to deliver a similar evaporation performance in the second 24-h test cycle (Fig. 4a), indicating that the 3D solar crystallizer can be easily regenerated and reused without noticeable change in real brine treatment performance. It was noticed that, when the solar light was turned off during the operation, the evaporation rate dropped to around 0.3 kg m^−2^ h^−1^ (Fig. 4a). However, the surface salt layer did not show any noticeable change even after the solar crystallizer was kept in dark for 12 h (Fig. S13), indicating the re-dissolution of the salt crystals was insignificant. In this case, we believe the salt re-dissolution was slowed down by the water evaporation in darkness, which limited the salt ions back diffusion from the evaporation substrate to the source brine. Upon turning on the light, the evaporation rate was recovered to the same level as the previous. Thus, the 3D solar crystallizer can operate continuously during day and night without special care while treating the concentrated SWRO brine, and the solid salts can be regularly removed from the device.

During the first 24-h operation, there were some small salt crystal grains cleared off the solar crystallizer’s wall by their own gravity (Fig. 4b). To further investigate salt defoliation behaviors during an un-interrupted operation of the solar crystallizer, another 48-h continuous experiment was conducted. As shown in Fig. 4f, a significant amount of the salt crystals fell off from the solar crystallizer’s wall by the end of the 48-h operation. For a better presentation, the solar crystallizer was then carefully taken out without significant shaking. The salt in the tray (right part of Fig. 4f) showed the self-defoliated salt produced during the operation.

To confirm the long-term operational stability, the solar crystallizer was continuously illuminated by the simulated sunlight (1 kW m^−2^) for 288 h in lab conditions to treat concentrated SWRO brine with NTA. There was no manual salt removal from the solar crystallizer and only the self-defoliated salt crystals were periodically collected during the operation. During the whole testing period, the performance of the solar crystallizer was largely stable and an average evaporation rate of 2.42 kg m^−2^ h^−1^ was achieved (Fig. 4g). After 288-h operation, the porosity of the QGF membrane was measured as 52.14%, only slightly lower than the raw QGF (68.20%). Both the stable performance and porosity measurement indicate that by adding NTA in the source brine, this solar crystallizer is able to deliver a long-term stable operation for highly concentrated SWRO brine without any manual removal of the salt from the solar crystallizer.

## Discussion

As mentioned above, the water evaporation performances of the same solar crystallizer is totally different when it is used to treat pure NaCl brines and real seawater brines. In the former case, the water evaporation keeps stable for a long period of time, while it drops quickly in the latter case, which is ascribed to the huge difference in salt crust structures in the two cases. The dense salt crust surface layer along with the salt crystals filling inside the QGF membrane in the case of the concentrated SWRO brine leads to the failure of the 3D crystallizer during the long term operations.

**Fig. 5:**
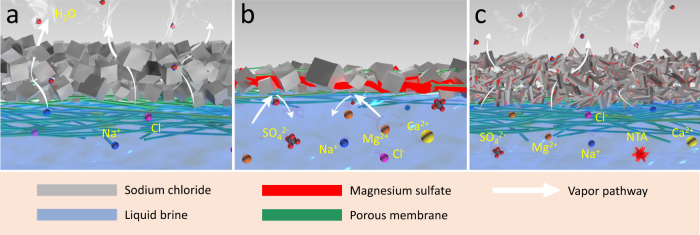
Schematic illustration of accumulated salt crystals. The salt layer when using solar crystallizer to treat **a** pure NaCl brine, **b** concentrated SWRO brine and **c** concentrated SWRO brine with NTA.

In order to ascertain the mechanism of forming the dense salt crust layers and inner pore filling crystals in the case of the real seawater brines, the crystallization behaviors of pure NaCl brine and concentrated SWRO brine were investigated and compared (the experimental details can be found in SI). The SEM images of the crystals formed by evaporating 20 wt% pure NaCl brine (Fig. S14) show individual and well separated cubic crystals only. For pure NaCl brine, as water is being removed from the evaporation surface, the temperature is lowered and the salt concentration is increased on the surface, which makes the salt crystals tend to precipitate on the outer surface and therefore it keeps the water channel inside the QGF membrane unblocked. Thus, the NaCl salt crust tends to growth along the out-of-plane direction to gradually increase its thickness and to maintain its porosity relatively consistent. The porosity of the salt crust formed by pure NaCl brine (20 wt%) is 19.3% based on mercury intrusion porosimetry measurement. As illustrated in Fig. 5a, the loosely packed pure NaCl salt crust layer permits liquid water and water vapor transport, which explains the stable water evaporation performance with pure NaCl brine.

**Fig. 6:**
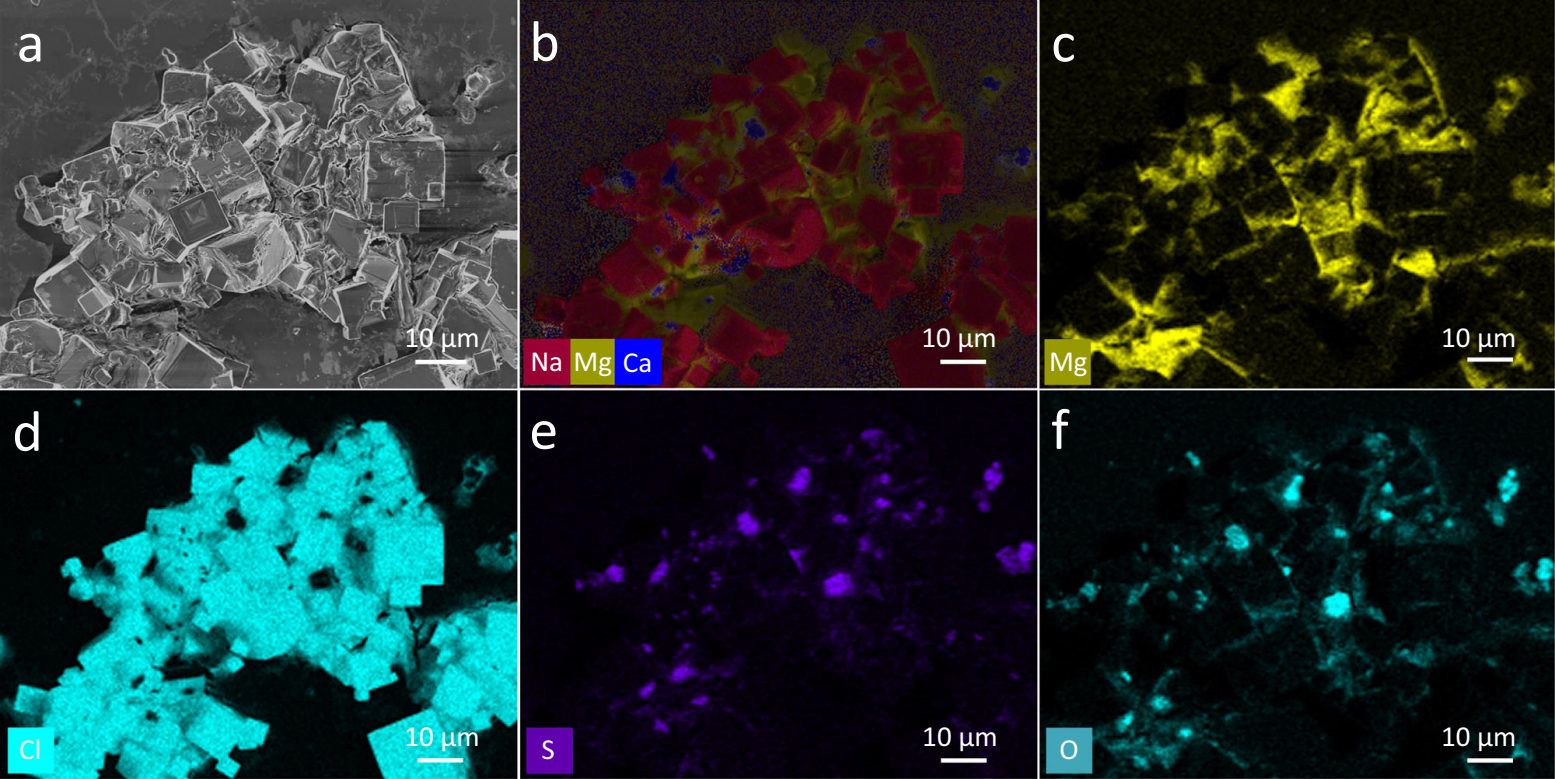
The morphology of the crystals from concentrated RO brine and the corresponding ion distribution. **a** SEM image of the crystals after completely drying concentrated RO waste brine directly on wafer and the corresponding EDS maps of **b** metal elements, **c** magnesium, **d** chloride, **e** sulfur, and **f** oxygen.

For the salt crystal aggregation formed from concentrated SWRO brine, the SEM images show mixed crystal phases, with cubic crystals dominating and the gap space between the cubes being filled with some wax-like substances (Fig. 6a). According to the elemental distribution maps by EDS analysis, the cubic crystals are mainly NaCl (Fig. 6b, d) with calcium sulfate crystals sparsely decorated on the NaCl cubic crystal surfaces as small islands (Fig. 6b, e, f). Very importantly, magnesium is identified in the wax-like substances among the cubic NaCl crystals, suggesting magnesium species should be responsible for forming the dense salts crust layer by sealing the gaps among the NaCl crystals. Based on our calculation (Supplementary Note S3), the volume of MgSO_4_·7H_2_O crystals can be 18.8% the total solid after a full evaporation of the concentrated SWRO brine. This result implies the MgSO_4_ inside the concentrated SWRO brine, when fully solidified as MgSO_4_·7H_2_O, could potentially fill the entire pore space existing around NaCl crystals (19.3%) and thus seal the water path to the water evaporation surface (Fig. 5b).

We hypothesize the followings as a possible mechanism for forming the surface dense crust layers and inner pore filling crystals in the case of the concentrated SWRO brines. As the salt precipitates during water evaporation, NaCl, as the most abundant species, precipitates dominantly while highly hydrous magnesium sulfate, as a minor species, precipitates only in the gap space among the cubic NaCl crystals. As the hydrous magnesium sulfate gradually fills the pore space among the NaCl crystals, at one point, a dense crust layer is formed on the surface, which renders the evaporation substrate unable to deliver water to the outer surface and thus water evaporation stops. At the same time, the suppressed evaporation with the real seawater brines leads to a much smaller negative temperature gradient from the outer to inner side of the QGF membrane and a decreased water flux from the inner side of the QGF membrane to its outer side, which benefits the salt ions back diffusion to the inner side. As a result, the inner side of the QGF membrane possesses the similar temperature and salt concentration to the outer surface, and thus promotes salt precipitation inside the membrane, unlike the case of pure NaCl brine.

To solve this problem, a salt crystallization inhibition strategy involving adding NTA into real SWRO was then proposed. The NTA makes cubic NaCl crystal become dendritic-shaped^46^. Predictably, it will lead the formation of a highly porous aggregation layer on the outer surface of QGF membrane. The large pore volume of the crust layer makes the available MgSO_4_·7H_2_O not able to entirely fill the pore space among the NaCl crystals, always maintaining sufficient pore space available for water transport to the evaporation surface.

**Fig. 7:**
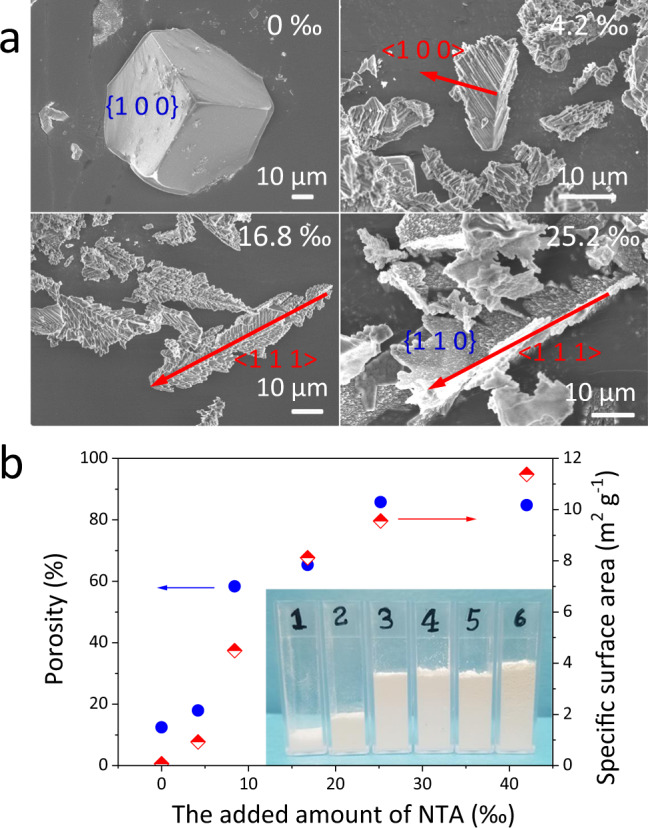
The physical property of the salt crystals in the presence of NTA. **a** The SEM images of the salt crystals in the presence of NTA; **b** the porosity and surface area of each salt samples; inset photo shows 0.4 g collected salt crystals formed from 12 wt% NaCl brine with (1) 0‰, (2) 4.2‰, (3) 8.4‰, (4) 16.8‰, (5) 25.2‰ and (6) 42.0‰ NTA in cuvette (the bottom side length: 10 mm).

In order to assess the efficacy of this strategy, we firstly investigated the effect of NTA on the crystallization behavior of NaCl crystal and its porosity. Twelve weight % of pure NaCl aqueous brines with different amount of NTA in it were used as source brines, and treated with the 3D solar crystallizer under otherwise the same conditions. At the end of 10-h operation, the morphologies of the salt crust layers show a strong dependence on the amount of NTA added in the NaCl brine, transitioning from hard salt layer to a fluffy pom-poms like structure (Fig. S15) with the NTA concentrations increasing from 0, 4.2, 8.4, 16.8, 25.2 to 42.0‰. However, the XRD patterns of these salt crusts (Fig. S16) did not show any significant change in their crystal phases, indicating that the presence of NTA did not affect the NaCl crystal type. However, the XRD patterns reveal that, with the co-presence of NTA in the brines along with NaCl, the growth of <1 0 0> crystal faces was hindered while <1 1 0> faces of NaCl crystals were preferred instead, indicated by the great change in the intensity ration between <1 0 0> diffraction peak and <1 1 0> diffraction peak^47^. Moreover, the SEM observation clearly demonstrates the shape of the NaCl crystals gradually turned from perfect cubic to dendritic shape (Fig. 7a and Fig. S17) (More details and discussions can be found in Supplementary Note S4). Consequently, it leads to a significant change of the physical properties of the salt crust layers. As shown in Fig. 5b, when the NTA concentrations in the source NaCl brines increased from 0, 8.4 to 42‰, the apparent densities of the NaCl crystal aggregates decreased from 0.97, 0.18 to 0.17 g/cm^3^, the porosity increased from 12.4, 58.3, to 84.8%, and the specific surface area increased from 0.08, 4.49, to 11.4 m^2^/g, respectively. All these results demonstrate that a small amount of NTA can significantly increase the porosity of NaCl crust layer by converting the shape of the NaCl crystal from cubic shape to dendritic shape.

Then the real seawater brines with different amount of NTA were used as source brines and treated by the solar crystallizer. As presented in Fig. 4e, the collected salt crystals also turned into dendritic shape in the presence of NTA. The porosity of the surface accumulated salt increased from 1.00 to 43.54% as the NTA concentrations were increased from 0 to 12.6 ‰ (Fig. S18). It should be pointed out that MgSO_4_ is quite uniformly distributed in the salt crust layer along the out-of-plane direction. The sodium-to-magnesium molar ratio of the surface crust after a 24-h operation is 12.6, which is consistent with that of the raw concentrated SWRO brine (13.1), suggesting that the magnesium would not selectively accumulate in the inner side nor the surface side. This feature is another reason for the excellent long-term operation stability of the 3D solar crystallizer as it ensures a stable porous structure of the salts crust layer. All these results strongly support the effectiveness of our proposed strategy of salt crystallization inhibition to control pore spaces of crystallized salt aggregates.

Note that the NTA is one of the various salt crystallization inhibitors to modify the morphology of NaCl crystal. Evaluation of the performance of other salt crystallization inhibitors is necessary in the future to further improve the performance of solar crystallizer while treating different brines with different compositions.

In addition, the scalability of solar crystallizer is also a critically important consideration. The solar crystallizer can be easily scaled up by enlarging its size and the brine treatment capacity of such a system can be multiplied by applying an array of the solar crystallizers. A few enlarged devices with varying magnification ratios were fabricated. Their similar evaporation rates prove the size magnification does not affect the performance significantly when they are used as a single unit. Then twelve solar crystallizers were assembled to form an array and the array was challenged to treat the highly concentrated real SWRO brine of 21.6 wt% outdoors. It turns out that the array delivered a high water evaporation rate of 48.0 kg m^−2^, showing its great potential for practical brine treatment. Under the field conditions, the solar incident angle, ambient temperature, relative humidity and natural wind all affect the performance of the solar crystallizer. Comprehensive evaluation of such effects is necessary for the practical application of the solar crystallizer towards practical ZLD brine treatment.

It has to be cautiously pointed out that the solar crystallizer at this point might not be very effective in treating large volume of the brine due to its large land requirement. However, comparing with the commercial ZLD system, which costs $250000 to over $2 million with the capacity of 5–100 m^3^ per day (https://www.samcotech.com/how-much-will-a-zero-liquid-discharge-system-cost-your-facility/), the solar crystallizer offers a low-barrier-of-entry option for treating brine with small to medium volume. For example, for a medium-sized chemical plant producing around 15 m^3^ concentrated brine every day, there will be an area of around 300 m^2^ solar crystallizer array plus interdevice space required to treat the brine with ZLD. Moreover, due to its simple setup, the array can be placed on the rooftop of factory buildings, which would further reduce its land footprint. The detailed discussions on the scalability of the solar crystallizer can be found in Supplementary Note S5. With ongoing work further improving the performance of the solar crystallizer, it is expected that the next-generation solar crystallizer will widen its application perspective.

The 3D solar crystallizer in conjunction with salt crystallization inhibitor in this work provides an opportunity for ZLD treatment of real seawater brines with long-term stability. The salt crystallization inhibitor modulates the morphology of salt crystals and solves the troublesome problem of salt scaling. This solar crystallizer with salt crystallization inhibitor enables a high water evaporation rate of 2.42 kg m^−2^ h^−1^ and the presence of a small amount of salt crystallization inhibitor makes the crystallizer’s regeneration possible, leading to long-term stable brine treatment operations. The high water evaporation rate of 48.0 kg m^−2^ per day achieved by the solar crystallizer array in outdoor field test also indicates its great potential for practical brine treatment. Further improvements in the performance of the solar crystallizer can be multi-faceted, including but not limited to applying artificial wind field to enhance environmental thermal energy harvesting, optimizing array assembly. The solar crystallizer is believed broadly applicable to various crystallization processes, such as salt recovery from waste brines, salt mineral extraction from salt lake, and thus is a meaningful contribution to the challenging problem of industrial brine treatment with ZLD.

## Methods

### Chemicals

Quartz glass fibrous filter membrane was purchased from Merck Millipore Ltd. (Catalogue Number AQFA8X105). Spectrally selective solar absorber, consisting of a cermet (ETA plus) coated on an aluminum sheet, was purchased from Alanod Solar. Aluminum tape was purchased from TED PELLA, INC. Sodium chloride was analytical reagents and purchased from Sigma-Aldrich. Nitrilotriacetic acid (NTA) was provided by Shandong Deshunyuan Petroleum Sci & Tech Co. Ltd. All chemicals were used as received without further purification. All aqueous brines were prepared using deionized water with a resistivity of 18.2 MΩ cm prepared by Millipore system.

### Characterizations

The surface morphology of the membranes and morphology of salt crystals were investigated by field-emission scanning electron microscopy (SEM, Zeiss Merlin). The powder X-ray diffraction patterns were recorded on a Bruker D8 Discover diffractometer using Cu Kα radiation (*λ* = 1.5418 Å) as X-ray source. The specific surface area was determined by a Micromeritics-TriStar II system, calculated using the Brunauer–Emmett–Teller method. The porosity of the salt crystal aggregates and QGF membrane samples was measured by Mercury Porosimeter (AutoPore IV, Micromeritics). The UV-Vis-NIR diffuse reflectance, transmittance and absorption spectra of the samples were recorded with a Perkin Elmer Lambda 950 spectrophotometer. All salt crystal sample was dried in a vacuum oven at 20 kPa and 313.15 K for 4 h before characterization.

### Preparation of real seawater brine samples

The raw seawater desalination brine (collected from RO plant inside King Abdullah University of Science and Technology in May 2018) was concentrated in a blast oven in 358.15 K and then filtrated by 0.45 μm PTFE filter. The seawater (collected from Red Sea near Thuwal, Saudi Arabia at January 2018) was concentrated using the same procedure. The detailed water quality of two real seawater brine samples was shown in Table S1. During the concentration process of RO waste brine, there were some white precipitates formed, which was removed by filtration. The XRD pattern of the precipitates (Fig. S19) indicates that their main compositions were calcium sulfate dehydrate (CaSO_4_·2H_2_O), which have a poor solubility in water. The salt concentration was 21.6 wt% for the concentrated SWRO brine and 16.8 wt% for the concentrated seawater.

### Solar evaporation and crystallization experiments

A lab-scale setup was built to evaluate the solar evaporation and crystallization performance of the solar crystallizer. A solar simulator (Oriel solar simulator) was used to provide solar radiation with a constant intensity of 1000 W m^−2^. A mass balance (ME204E, Mettler Toledo) was used to record the water mass change. The temperature distribution was monitored by an IR camera (A600-series, FLIR). The evaporation rates of the solar crystallizer with different heights when treating pure water were measured under one sun illumination for 3 h. The evaporation rates of the solar crystallizer when treating various brine sources under one sun illumination were recorded. When treating concentrated SWRO brine, there was no manual salt removal from the solar crystallizer unless otherwise specified. All lab measurements were conducted at an ambient temperature of 21–22 °C with humidity of ~60%. The outdoor field tests were conducted on the rooftop of a housing unit inside KAUST campus during August 21–25, 2020.

## Supplementary information

Supplementary Information

Supplementary Video

## Data Availability

The authors declare that the data supporting the findings of this study are available from the authors upon request.
