# Supplementary material for: Designing a next generation solar crystallizer for real seawater brine treatment with zero liquid discharge
Source: Nat Commun. 2021 Feb 12;12:998. doi: 10.1038/s41467-021-21124-4 (PMC7881092; doi:10.1038/s41467-021-21124-4)
Supplement: Supplementary file 1 — Supplementary Information [file 41467_2021_21124_MOESM1_ESM.pdf]

Supplementary Information

Solar Crystallizer: A New Strategy for Real Seawater Brine Treatment with Zero Liquid Discharge

Chenlin Zhang[1], *Yusuf Shi*[1], *Le Shi*[1], *Hongxia Li*[2], *Renyuan Li*[1], *Seunghyun Hong*[1], *Sifei Zhuo*[1], *Tiejun Zhang*[2] *and Peng Wang*[1,3]*

[1] Water Desalination and Reuse Center, Division of Biological and Environmental Science and Engineering, King Abdullah University of Science and Technology, Thuwal, 23955-6900, Saudi Arabia.

[2] Department of Mechanical Engineering, Masdar Institute, Khalifa University of Science and Technology, PO Box 54224, Abu Dhabi, United Arab Emirates.

[3] Department of Civil and Environmental Engineering, The Hong Kong Polytechnic University, Hong Kong, China

[*] E-mail: peng.wang@kaust.edu.sa

Supplementary Figures and Tables

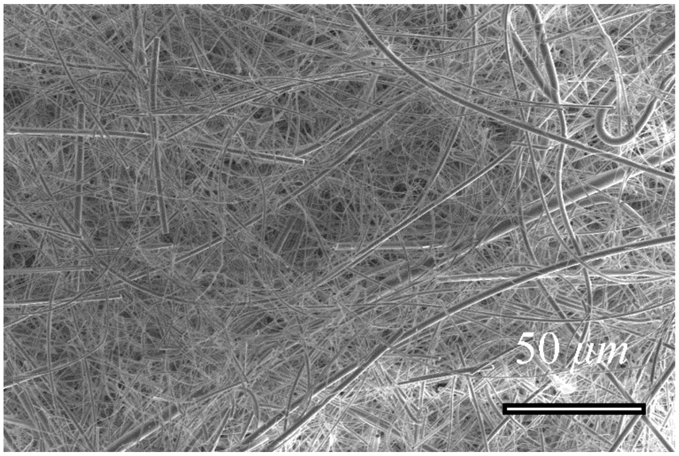

Figure S1. SEM image of the pristine quartz glass fibrous membrane. The membrane had a porous structure.

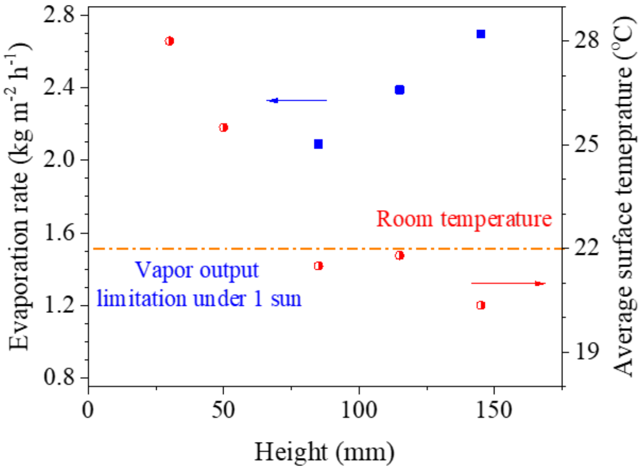

Figure S2. The evaporation performance of the solar crystallizers with different heights under one sun illumination along with its average surface temperature (pure water was used for measurement). When its average surface temperature was blow the room temperature, the evaporation rate would be over the vapor output limitation under 1 sun.

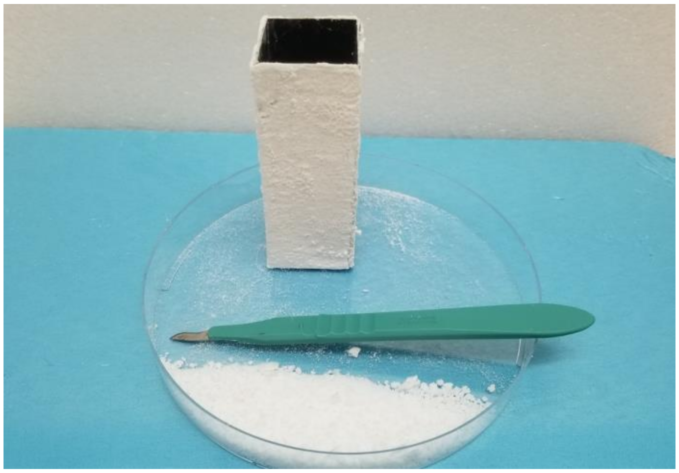

Figure S3. Photo images of the solar crystallizer after removing accumulated salt when treating pure 24 wt% NaCl brine. The crust layer of NaCl crystal balls could be removed by a stainless steel scraper.

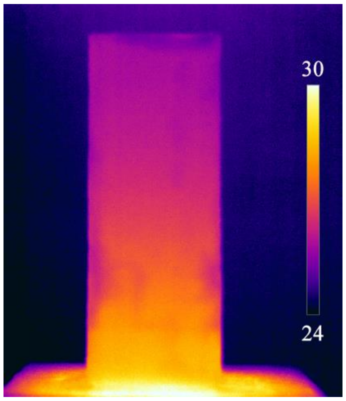

Figure S4. The IR image of a solar crystallizer after 24-hour operation when treating pure 24 wt% NaCl brine (unit: ◦C). The solar crystallizer showed a relatively uniform temperature distribution.

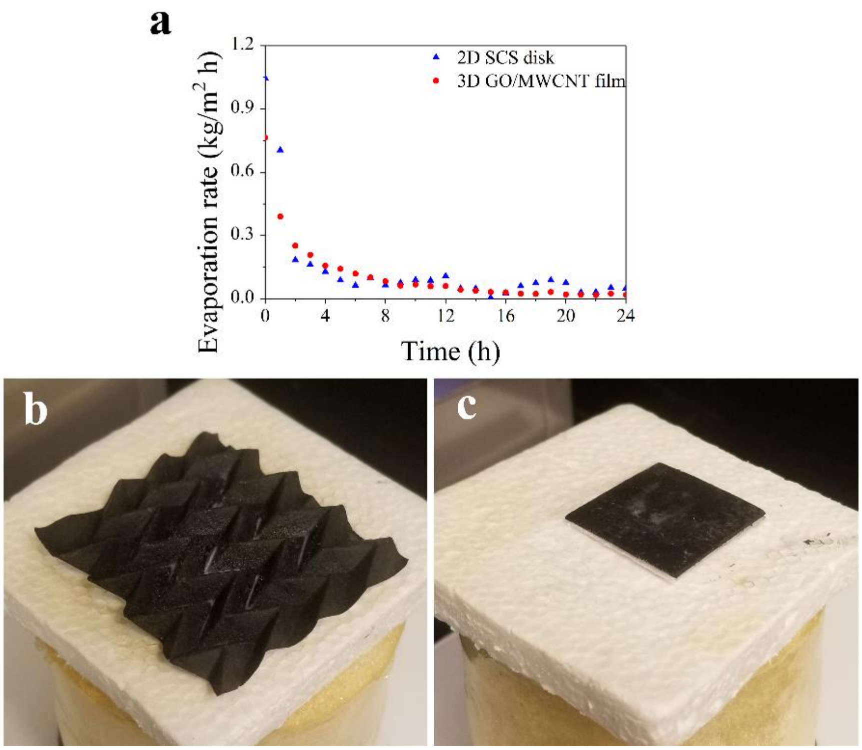

Figure S5. The crystallization behavior of real seawater brine in solar crystallizers. (a) The solar-driven water evaporation rate decay of 2D silica/carbon/silica tri-layered coaxial fibrous membrane (SCS) disk and 3D graphene oxide/ multi-walled carbon nanotube coated cellulose membrane (GO/MWCNT) film while treating concentrated SWRO brine (21.6 wt%). The image of the photothermal materials after 24-hour operation by (b) 3D GO/MWCNT film and (c) 2D SCS disk. (Note: In these two cases, when the water evaporation rates had dropped to almost zero, the crystallizer surfaces appeared quite black, indicating the light absorption of these crystallizers was not significantly degraded by the precipitated salt layer.)

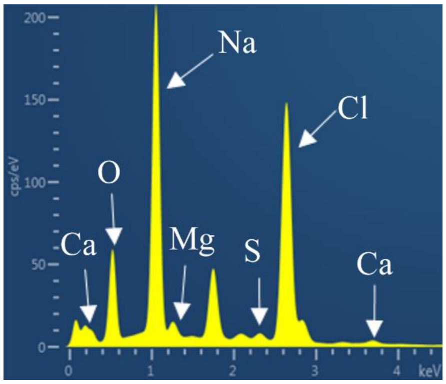

Figure S6. The EDS analysis of the salt crust layer formed from the concentrated SWRO brine (21.6 wt%). This salt crust layer contained sodium, chlorine, magnesium, calcium and sulfur elements.

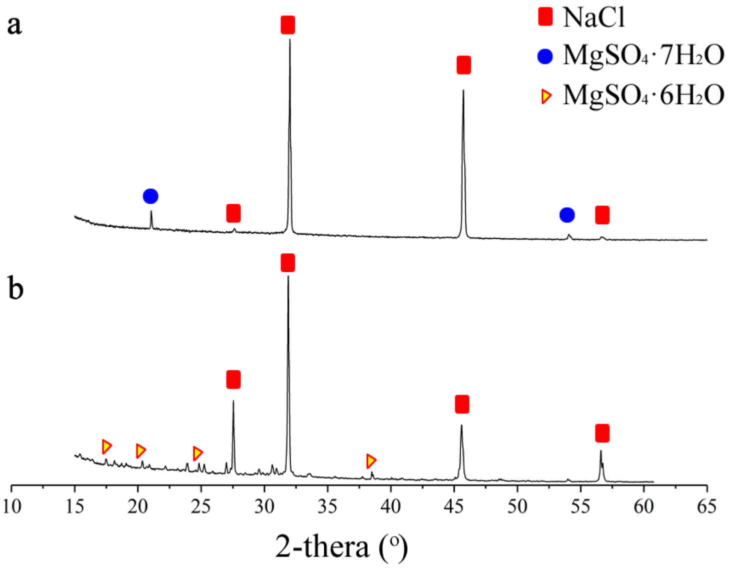

Figure S7. The XRD pattern of (a) the wet salt crust layer and (b) the dry salt crust layer after 24-hour operation with the concentrated SWRO brine (21.6 wt%).

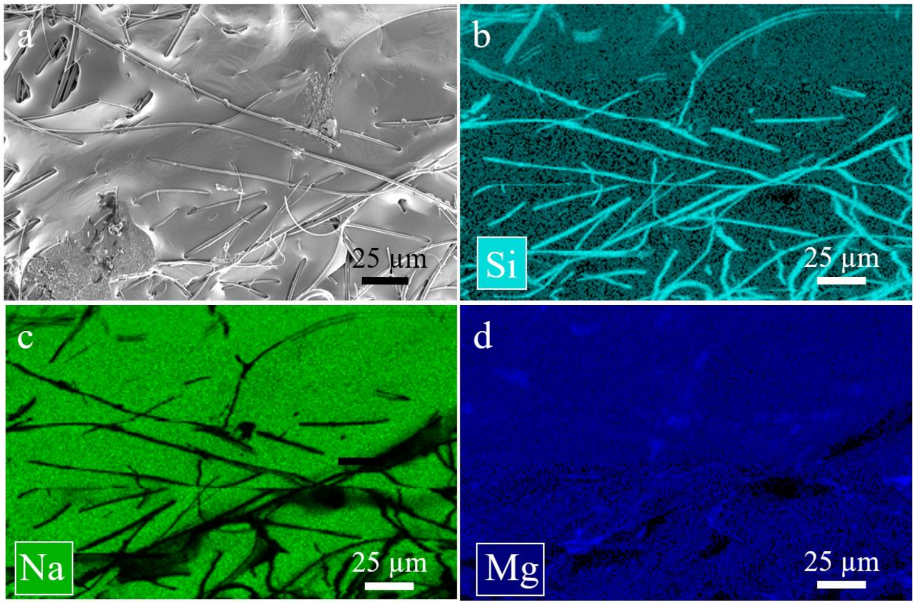

Figure S8. The morphology of the scaling inside QGF membrane and corresponding ion distribution after treating concentrated SWRO brine (21.6 wt%). (a) SEM image of scaling and corresponding EDS mappings of (b) silica, (c) sodium and (d) magnesium.

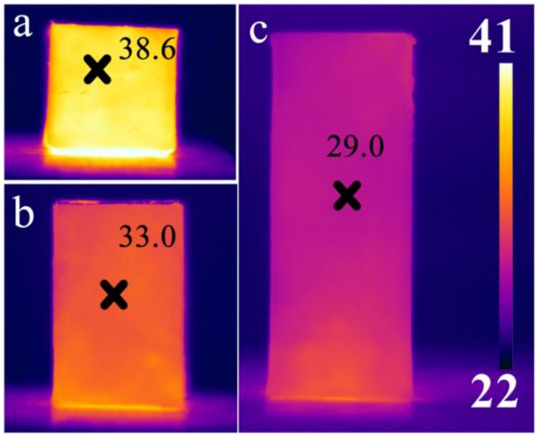

Figure S9. The IR images of the solar crystallizers with different heights in dry state under one-sun illumination (unit: °C). The temperature distribution of the solar crystallizer with height of (a) 35 mm, (b) 50 mm and (c) 85 mm.

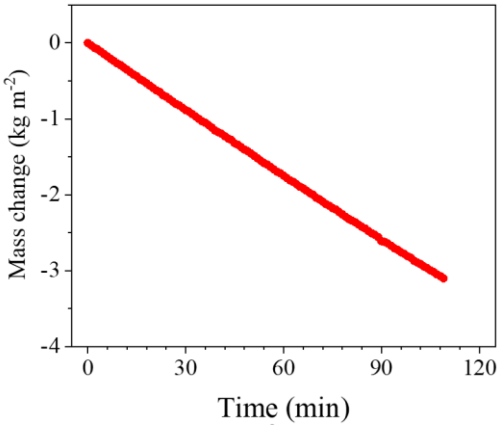

Figure S10. The mass change curve of 21.4 wt% pure NaCl brine under one sun illumination. The corresponding water evaporation rate was 1.71 kg m^{-2} h^{-1}.

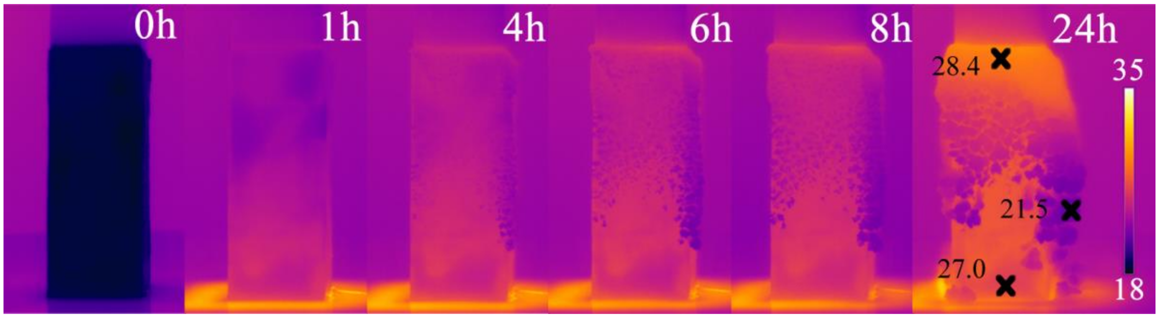

Figure S11. The IR images of the solar crystallizer while treating concentrated SWRO brine (21.6 wt%) with nitrilotriacetic acid (NTA). After 24 hours, the highest surface temperature of the crystallizer was 28.4 ˚C under solar radiation.

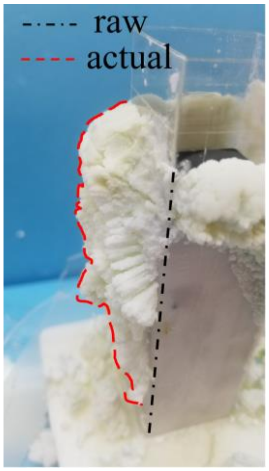

Figure S12. The actual evaporation surface of the solar crystallizer after forming salt crystals. The black line represents the raw edge and the red line represents the actual edge after treating concentrated SWRO brine for 24 hours.

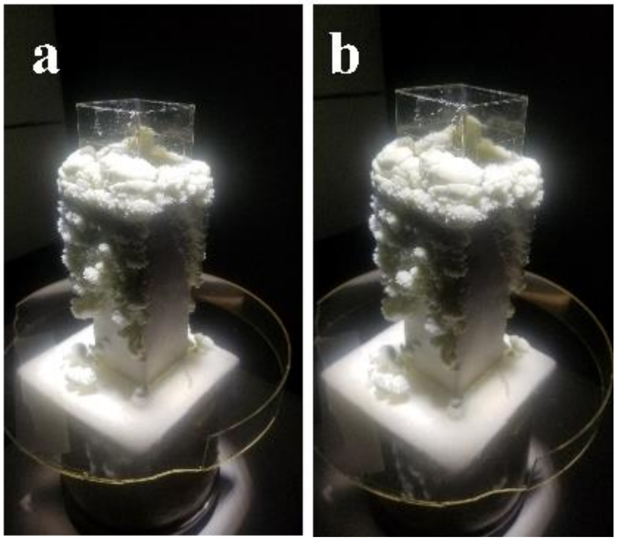

Figure S13. The photos of the solar crystallizer with accumulated salt crystals (a) before and (b) after 12 hours period without illumination.

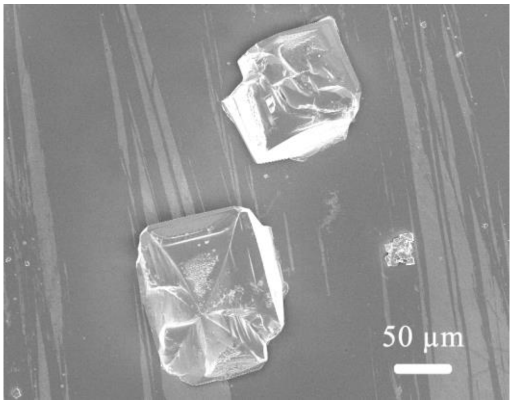

Figure S14. The SEM image of the salt crystals after pure 20% NaCl solution drying on the wafer. The cubic crystals were well separated.

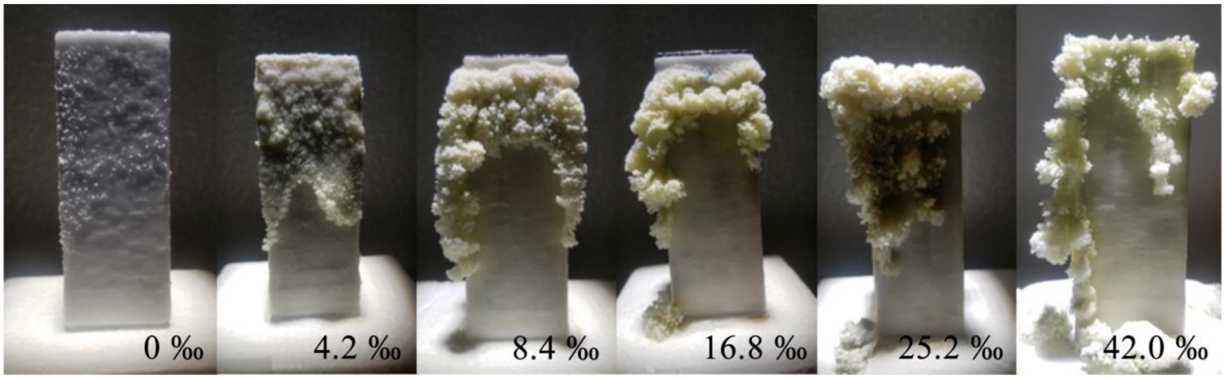

Figure S15. Photo images of the salt crust layer formed from 12 wt% NaCl brine with different amount of NTA. When the NTA concentrations increased, the morphologies of the salt crust layers turned from hard salt layer to a fluffy pom-poms like structure.

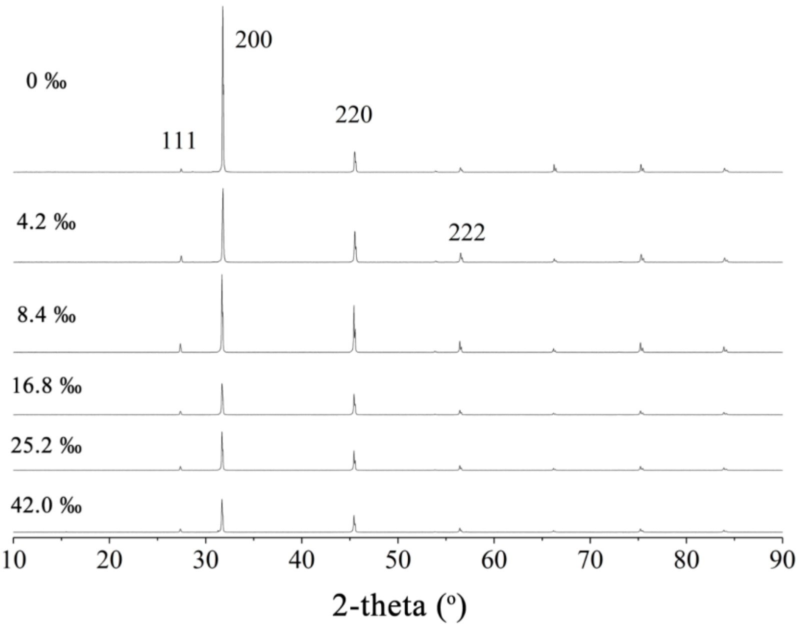

Figure S16. The XRD patterns of the salt samples in the presence of different amount of NTA. These salt samples were the same in their crystal phases

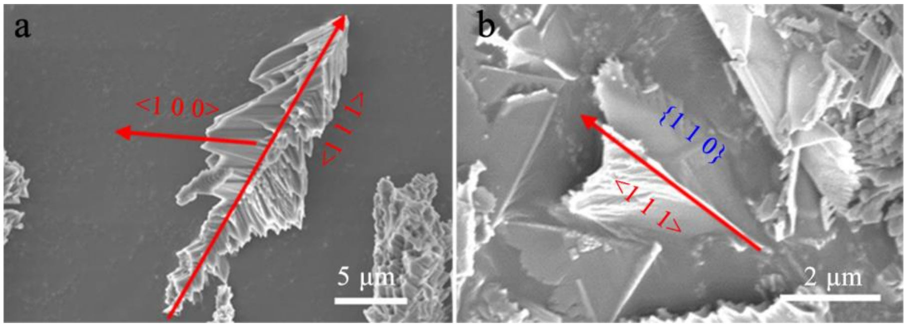

Figure S17. The morphologies of the salt crystals formed from 12 wt% NaCl brine with different NTA amount. The SEM images of salt crystals after adding (a) 16.8‰ and (b) 42.0‰ of NTA into brine.

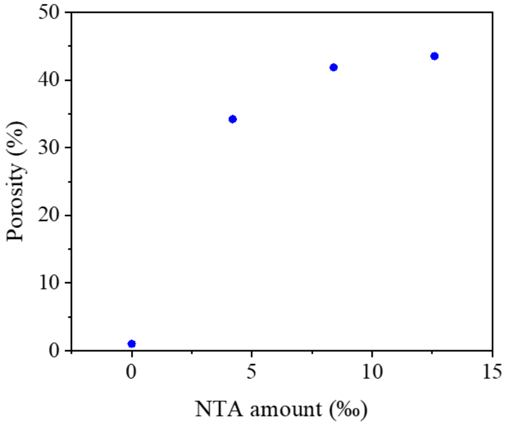

Figure S18. The porosity of the collected salt crystals formed from the concentrated SWRO brine (21.6 wt%) with different amount of NTA. The porosity of the collected salt crystals increased from 1.00 to 43.54 % as the NTA concentrations were increased from 0 to 12.6 ‰

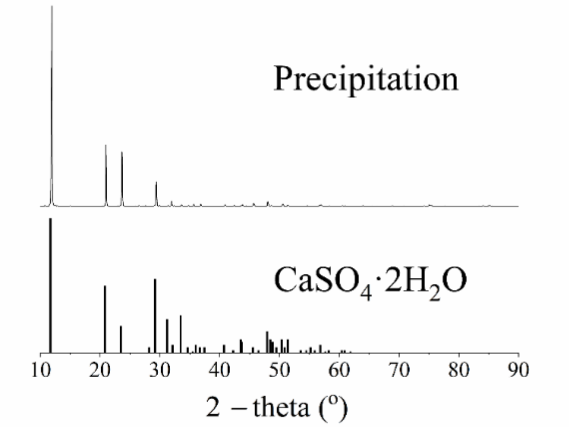

Figure S19. The XRD pattern of the precipitates collected during the concentration of SWRO brine (21.6 wt%). Their main compositions were calcium sulfate dehydrate ($CaSO_4 \cdot 2H_2O$).

Table S1. The ion composition of brine samples.

Ion composition (g/L)	Concentrated SWRO brine	Concentrated seawater
Na^+	63.8	57.0
K^+	2.8	0.8
Ca^{2+}	0.8	1.02
Mg^{2+}	8.6	3.0
Cl^-	128.9	88.9
SO_4^{2-}	10.9	12.3

Supplementary Note S1. Solar crystallization process inside the solar crystallizer.

(1) Solar-to-vapor efficiency.

In this work, the solar absorptance[1] of the solar crystallizer with different heights can be determined using the following equation:

$$\alpha = \frac{\int_{280}^{2500} I(\lambda)A(\lambda)d\lambda}{\int_{280}^{2500} I(\lambda)d\lambda} \qquad \text{(Equation S1)}$$

where $I(\lambda)$ and $A(\lambda)$ are the light intensity function of the solar spectrum and the absorption function of each sample at different wavelength (λ). The transmittance was assumed to be zero, which agreed with the actual situation of the devices. Light intensity function of the solar spectrum and the UV-vis-NIR spectra of each solar crystallizers were shown in Figure 2a. The solar absorption for the flat SSA material was 0.94. The 3D structures exhibit a higher solar absorption of 0.96 for 30 mm height, 0.98 for 50 mm height and 0.99 for 85 mm height. The solar absorptance of the solar crystallizers with height over 85 mm were not measured due to the size limitation of the lab instruments and their solar absorptance can be regarded as 0.99.

The apparent evaporation efficiency (η)[1-3] is calculated *via* the following formula as in most literature reports:

$$\eta = \dot{m} \times (L_v + Q)/P_{in} \qquad \text{(Equation S2)}$$

where $\dot{m}$ is the apparent water evaporation rate, L_v and Q are the latent heat and sensible heat of water evaporation process, and P_{in} is the power of the incident simulated sunlight beam, which is 1 kW m^{-2} in our experiment.

When these crystallizers were exposed under one-sun illumination, the apparent evaporation rate of pure water 2.09 kg m^{-2} h^{-1} for 85 mm height. The net water evaporation rate was 1.43 kg m^{-2} h^{-1} in this case, calculated as the difference between the water evaporation rate recorded under light and that recorded in dark (Figure S20a). The latent heat L_v is dependent on the temperature (T_i) of the water/air interface where the vaporization occurs, which can be calculated by an empirical formula: $L_v(T_i) = 1.91846\times10^6[T_i/(T_i-33.91)]^2$ according the literature[4]. The sensible heat Q can be calculated by the equation: $Q = c\ (T_i - T_s)$, where c is specific heat of water, 4.2×10^3 J kg^{-1} K^{-1} and T_s is the temperature of the source water, which is 21.0°C in this case. The average temperature of the water/air interface was used as T_i in this case (Figure S20b). Therefore, under one sun illumination, the solar crystallizer with height of 85 mm exhibited an apparent solar evaporation efficiency of 138.5% and a net solar evaporation efficiency of 94.3%.

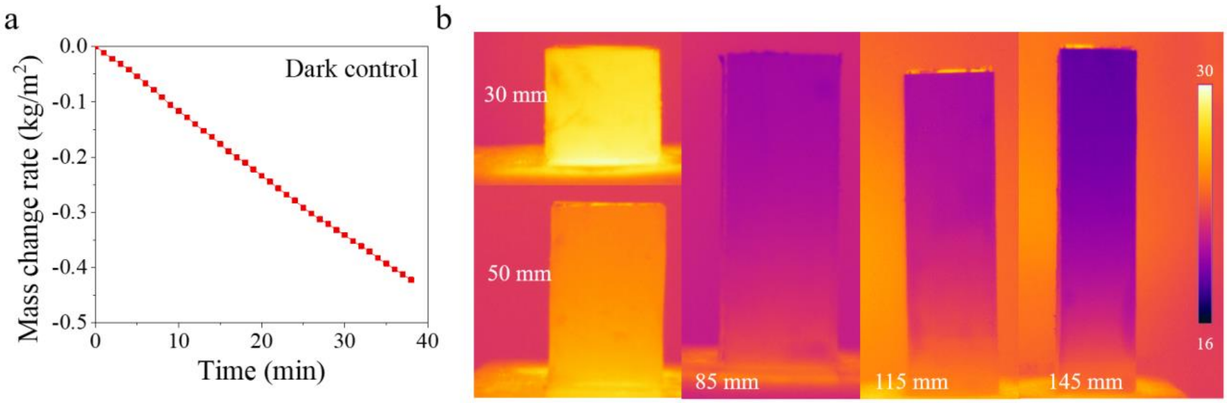

Figure S20. The evaporaiton performnace of the solar crystallizer with height of 85 mm under dark condition and the surface temeprature distribution of solar crystallizer with different heights under one sun illumination. (a) The mass changes of solar crystallizer with height of 85 mm under dark condition; (b) the IR images of the solar crystallizers with different height in wet state under one sun illumination.

(2) Concentration of the feed brine.

As illustrated in Figure S21a, the feed water is wicked and moves upward from the source brine reservoir via a porous fabric and then spreads over the outer wall of the crystallizer driven by capillary force. Under solar illumination, the water evaporation and salt crystallization take place only on the outer wall surfaces of the device. To estimate the change of the concentration of the source brine inside the source brine reservoir, the transport of salt between the solar crystallizer and brine reservoir is analyzed. As presented in Figure S21b, the salt transport can be divided into advection and diffusion. In the solar crystallizer design, the dissolved salt is transported out of the source brine along with water driven by capillary force (i.e., advection). At the same time, due to the evaporation on the outer surfaces of the crystallizer wall, the brine concentration in the crystallizer (C_1) is higher than that in the feed water inside the brine source reservoir (C_0), which driven the salt ions to diffuse from the crystallizer back to the brine source reservoir due to the concentration gradient.

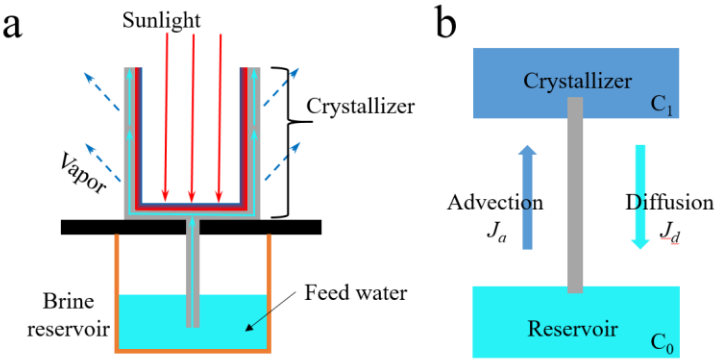

Figure S21. The schematic of mass transport inside solar crystallizer. (a) The schematic of water transport inside the solar crystallizer; (b) the schematic of salt transport inside the solar crystallizer.

The mass transportation rate of salt via advection (J_a) can be calculated as follows:

$$J_a = \frac{\dot{m}}{(1 - C_0)} C_0 \qquad \text{(Equation S3)}$$

where $\dot{m}$ is the apparent water evaporation rate, and C_0 is is the brine concentration of feed water.

The mass transportation rate of salt via back diffusion (J_d) can be calculated as follows:

$$J_d = \frac{D_{NaCl} \rho_w A}{l_w} (C_1 - C_0) \qquad \text{(Equation S4)}$$

Where D_{NaCl} is the diffusion coefficient of NaCl in water (1.99×10^{-9} m^2/s), $\boldsymbol{\rho}_w$ is the density of water (1000 kg m^{-3}), A is the cross-section area of capillary strip connecting crystallizer and brine reservoir (0.01 m×0.002 m), and l_w is the length of the strip for diffusion (0.02 m).

In this work, the starting concentration of the feed water (C_0) was 21.6 wt% and the brine concentration in the crystallizer C_1 was assumed as 36 wt% (the saturation concentration of NaCl brine; the actual concentration should lower than this). Based on Equation S4, the salt back diffusion should be lower than 1.03×10^{-3} g h^{-1}. At the same time, the evaporation rate of the solar crystallizer while treating pure NaCl brine was 1.71 kg m^{-2} h^{-1} under one sun illumination, leading to an advection salt transport of 4.52 g $h^{-1}\times10^{-1}$ g h^{-1} from the source reservoir to the solar crystallizer. Given the fact that there are around 200 grams of the source brine in the reservoir and the very large difference between the salt diffusion and advection, the concentration of the source brine inside the brine reservoir can be considered negligible.

(3) Capillary height.

The solar crystallizer presented in this work only utilizes capillary force to lift brine from the source reservoir to the evaporation surface on the top. The capillary height (h) can be calculated by using Equation S5:

$$h = \frac{2\gamma \cos\theta}{R\rho g} \qquad \text{(Equation S5)}$$

Where $\boldsymbol{\gamma}$ is the surface tension of brine (77.89 ± 0.03 mN for 21.6 wt% brine with NTA), $\boldsymbol{\theta}$ is the contact angle (0°, due to the superhydrophilic property of quartz glass fibrous filter membrane), R is the average pore size of the quartz glass fibrous filter membrane used in this work (12.75 µm, measured by mercury porosimetry), $\boldsymbol{\rho}$ is the density of the brine (1.16 g mL^{-1} for 21.6 wt% brine with NTA) and g is the acceleration of gravity.

The calculation indicates that the capillary height limit is 1.07 m in this work. However, this will not be a problem in practical application because a height of 1 m is sufficient to produce satisfactory water evaporation performance. Furthermore, other means of water delivery to evaporation surfaces can be considered when necessary, for example, pumping.

Supplementary Note S2. COMSOL model.

A COMSOL model was setup to simulate the evaporation process of the solar crystallizer. In the model, the evaporation is regulated by both heat and mass transfer balance. From an energy perspective, the vapor flux can be expressed as the following (Equation S6):

$$\dot{m}L_v = P_{in} + Q_{cov} + Q_{rad} \qquad \text{(Equation S6)}$$

where $\dot{m}$ represents the vapor flux normalized by illumination area, L_v is the enthalpy of water vaporization, P_{in} represents the solar energy input, and Q_{con} and Q_{rad} represent the energy exchange via air convection and radiation, respectively. The Q_{con} can be calculated by the heat transfer equation (Equation S7):

$$Q_{con} = \rho C_p \frac{\partial T}{\partial t} + \nabla \cdot (-k\nabla T) + \rho C_p \mathbf{u} \cdot \nabla T \qquad \text{(Equation S7)}$$

Where $\boldsymbol{\rho}$ is the density of air, C_p is the specific heat capacity of air, T is the temperature of air, t is the time, k is the thermal conductivity of air, and $\mathbf{u}$ represents the velocity field of air caused by natural convection. In natural convection, the temperature difference produces the air density variation that drives the buoyant flow. Here, the term ($\frac{\partial T}{\partial t}$) is zero as only a steady state is studied.

The Q_{rad} follows the Stefan-Boltzmann law:

$$Q_{rad} = A\varepsilon\sigma({T_{amb}}^4 - {T_s}^4) \qquad \text{(Equation S8)}$$

Where A is the radiating surface area, ε is the Stefan-Boltzmann constant and σ is the emissivity of the radiating surface, T_{amb} is the ambient temperature and T_s is the radiating surface temperature.

According to thermodynamics, the vapor is generated at the water-air interface, and then transports into ambient environment under the gradient of vapor concentration. Therefore, the evaporation process is modelled as Equation S9:

$$\frac{\partial C_v}{\partial t} + \nabla \cdot (-D\nabla C_v) + \mathbf{u} \cdot \nabla C_v = \frac{G}{M_v} \qquad \text{(Equation S9)}$$

where M_v is the molecule weight of water, C_v is the vapor concentration, D is the diffusion coefficient of water vapor in air and G is the source term.

In the above governing equation, there are two contributing factors to vapor transport, vapor concentration gradient (diffusion,$\nabla \cdot (-D\nabla C_v)$) and air flow (mixing,$\mathbf{u} \cdot \nabla C_v$). On the water-air interface, the vapor concentration is assumed as saturated concentration C_{sat}, determined by the saturated vapor pressure p_{sat} under its surface temperature T:

$$C_{vat} = \frac{p_{sat}}{R_g T} \qquad \text{(Equation S10)}$$

In the simulation, the solar crystallizer is simplified as a 2D configuration (Figure S22a). By using the symmetric model, only half of the crystallizer is simulated. The ambient environment has the temperature of 22 ℃ and relative humidity of 60%. The solar irradiation is 1000 W/m^2, and is fully absorbed as heat input. The evaporation surface is set as wetted surface having saturated vapor concentration, and correspondingly the boundary heat source is chosen as latent heat source. By performing the coupled heat and mass transfer simulations, we can directly obtain the temperature, velocity and vapor concentration profile for the whole calculation domain. Figure S22b is an example showing the simulation results for a crystallizer design with 31 mm width and 30 mm height.

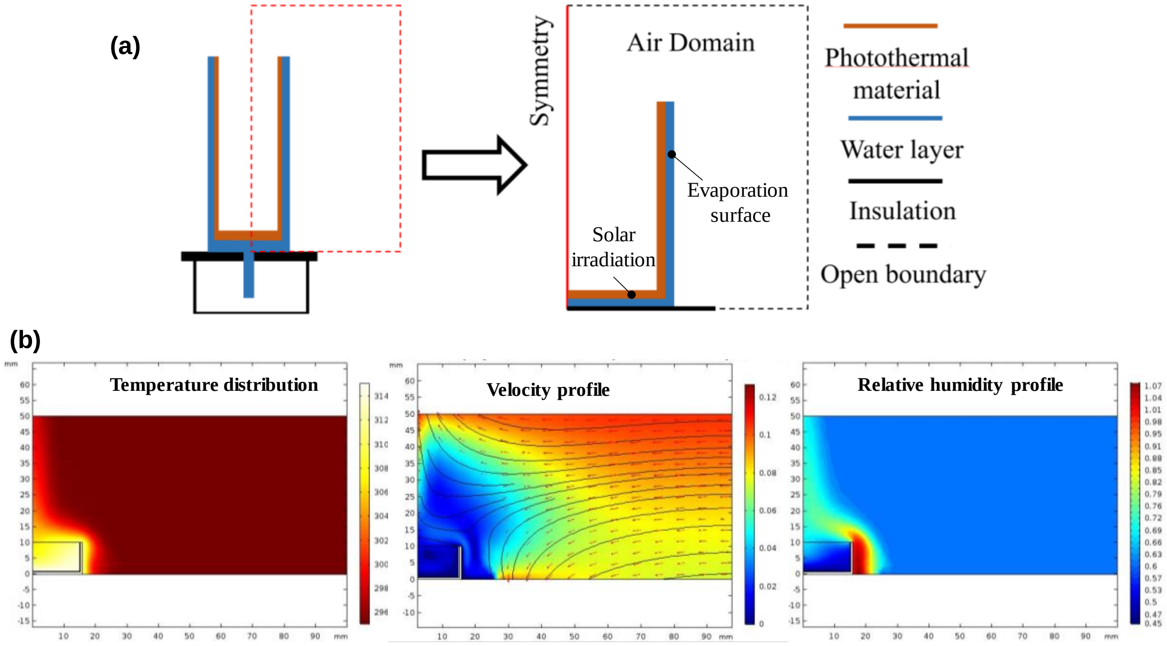

Figure S22. The configuration of the model and simulation results. (a) The 2D configuration of solar crystallizer; (b) the temperature, velocity and relative humidity profiles obtained from direct evaporation modeling.

To evaluate the vapor generation performance for solar crystallizer with different height (fixed width of 31 mm), we calculated the average temperature of the evaporation surface and evaporation rate. The results in Figure S23 show that once the solar crystallizer is over a certain height, the average temperature of the evaporation surface becomes lower than the ambient environment, forcing the environmental thermal energy flow into the solar crystallizer. Therefore, the performance of the solar crystallizer is well above the theoretical limit of vapor output under one sun (~ 1.52 kg m^{-2} h^{-1}) and increases linearly with the height of solar crystallizer, in an agreement with the actual evaporation rates measured in lab (Figure S2).

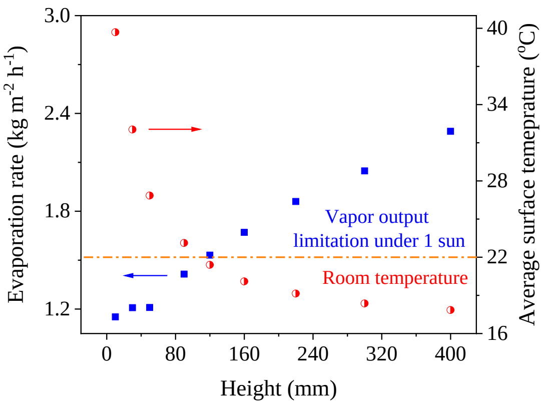

Figure S23. The simulation results of the model. The simulated evaporation rates and average surface temperature of the solar crystallizer with different height (fixed width of 31 mm).

Supplementary Note S3. Mechanism of scaling caused by magnesium sulfate.

In order to investigate the mechanism of the formation of a dense crust layer with the concentrated real brine, a small drop of the concentrated SWRO brine and pure 20 wt% NaCl brine were dropped onto a silica wafer and then dried at room temperature and ~60% relative humidity. The structure of the salt crystal aggregates was investigated using SEM observation with EDS elemental mapping analysis.

According to the Table S1, in the concentrated SWRO brine, the concentrations of $MgSO_4$ and NaCl were 29.2 g/L and 162.3 g/L respectively. $MgSO_4$ has a variety of hydrates. XRD analysis identified $MgSO_4•7H_2O$ as the major species inside the wet salt crust layer. The density of NaCl crystal[5] is 2.17 g/cm^3 while that of $MgSO_4•7H_2O$ crystal is 1.68 g/cm^3. Based on this, the volume fraction of $MgSO_4•7H_2O$ after crystallization of the brine was calculated. Other salts due to their small concentrations were not considered in the calculation.

The volume of each salt can be calculated as follows:

$$V = \frac{m}{\rho} \qquad \text{(Equation S11)}$$

where V is the volume of salt, m is the mass of salt and ρ is the density of salt.

The volume of $MgSO_4•7H_2O$ and NaCl was 17.4 cm^3 and 75.0 cm^3 after 1L concentrated SWRO brine was crystallized, respectively. The volume fraction of $MgSO_4$ was 18.8%, which was only slightly lower than the porosity of pure NaCl crystal (19.3%). This means the $MgSO_4$ inside brine can potentially fill the gap space and block the evaporation substrate.

Supplementary Note S4. The effect of NTA.

NaCl aqueous solutions with 12 wt% NaCl and different amount of NTA were used as source waters for testing, and they were treated on the 3D solar crystallizer. The morphology of the salts crust layer after 10-hour illumination gradually changed from dense glass-like layer to a fluffy pom-poms like structure (Figure S12) as the added amount of NTA increased from 0 to 4.2, 8.4, 16.8, 25.2 and 42.0‰. Some of the dendritic crystals even fell off the surfaces under their gravity. The salts crusts were removed from the solar crystallizer and dried by vacuum oven at 20 kPa and 313.15 K for 4 hours for characterization. The XRD patterns of these samples (Figure S12) did not show any change in crystal phase, indicating the presence of

NTA did not affect the crystal phase. However, the intensity ratio of the <2 0 0> Bragg diffraction to the <2 2 0> Bragg diffraction was on the decline as the NTA amount was increased. These results can be explained by that the growth of <1 0 0> crystal facets[6] was hindered and the <1 1 0> faces[6] of NaCl crystals were preferred after adding more NTA. The decrease of intensities of all diffraction peaks after adding more than 16.8‰ NTA was caused by the much smaller crystal size under the effect of NTA.

The SEM observation clearly demonstrates the salt crystals growth mode changes after adding NTA (Figure 7a and Figure S13). The pure NaCl aqueous solution produced cubic NaCl crystals with surfaces composed by <1 1 0> faces. After adding NTA, the growth of <1 0 0> face was suppressed, the <1 1 0> faces trended to develop well and the crystal tended to grow in the direction parallel to the <1 1 1> direction. Consequently, the shape of the crystals eventually turned from perfect cubic to dendritic, resulting in a significant change of their physical properties.

Supplementary Note S5. Discussion on scalability of solar crystallizer.

Scalability is a critically important consideration for the solar crystallizer. The solar crystallizer can be easily scaled up by enlarging its size and the brine treatment performance can be multiplied by applying an array of the solar crystallizers. Here, the scalability of solar crystallizer are presented in these two aspects. In addition, the influence of the solar incident angle and ambient conditions on the device performance are also discussed in this section.

(1) The performance of the enlarged devices.

The scaling up of the solar crystallizer was achieved by magnifying the whole device proportionally, namely with a fixed height to width ratio. A few enlarged devices with varying magnification ratio (85mm-wall height/31mm-bottom width was adopted as the original size based on which the magnification ratio were calculated) were fabricated. As presented in Figure S24a, the enlarged solar crystallizers all show the water evaporation performances similar to the original one.

Then we turned to COMSOL model to simulate single enlarged device performance at higher magnification (160mm/31mm was adopted as original size) and the simulation results are presented in Figure S24b. As seen, the performance of the single enlarged solar crystallizer (1.67 kg m^{-2} h^{-1}) is almost unchanged even after being magnified by 8 times (1280mm/248mm).

These experimental and simulation results all show similar average surface temperature of these enlarged devices (Figure S24a and b), indicating their similar capacity of environmental heat harvesting.

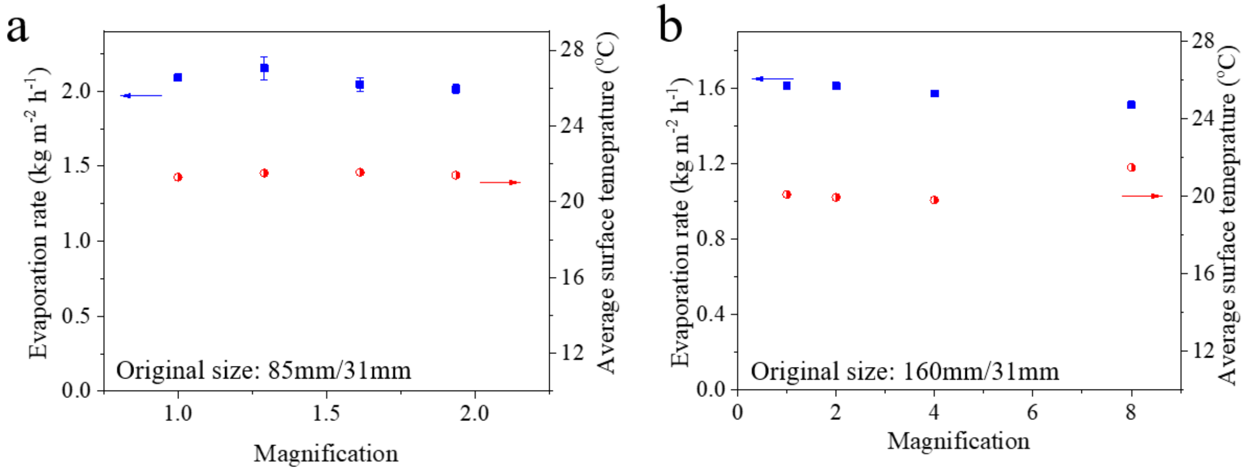

Figure S24. The water evaporation rate and average surface temperatures of single solar crystallizer with different magnification ratios (pure water was used for the performance evaluation indoor). (a) The experimental results; (b) the simulation results.

(2) The performance of the device array.

As presented in Figure S25a, twelve solar crystallizers (85 mm/31 mm) were assembled to form an array (with inter-device space of 39 mm uniformly), which was tested on the rooftop of a housing unit inside KAUST campus during August 21-25, 2020. One single solar crystallizer of the same size was setup besides the array as a reference. The highly concentrated SWRO brine of 21.6 wt% was used as source brine. The real-time evaporation rates were recorded and presented in Figure R25b while the temperature, relative humidity and natural wind velocity were recorded and presented in Figure S25c and d. It turns out that the average evaporation rates of the single solar crystallizer and the device array were 60.7 kg m^{-2} and 48.0 kg m^{-2} per day (including nighttime), respectively. As seen, the performance of the solar crystallizer decreased with the array setup due to hindered environmental heat harvesting as predicted by the reviewer. However, examining the real-time water evaporation rates in Figure S26b reveals something interesting and exciting. Taking the result of Aug 23rd as an example, the average water evaporation rate of the solar crystallizer array during the day time (08:00 to 16:00) was 5.5 kg m^{-2} h^{-1}, much higher than these measured in lab conditions. This indicates the solar crystallizer array can still gain considerable environmental thermal energy under field conditions, which will be explained later.

It is worth pointing out that, for real application, the total land area is larger than the required solar illumination area due to the presence of gap space among solar crystallizers if device array is to be used. In our outdoor field tests with the device array, the effective solar illumination area of the solar crystallizer

array was 115 cm^2 while the total projected area of the entire array was 588 cm^2. When normalized by the total projected area, the water evaporation rate of the solar crystallizer array was 9.4 kg m^{-2} per day. While we believe the performance of the current design can be further significantly improved with device and array optimization and introduction of artificial wind field, among others, it compares favorably against open water bodies whose water evaporation rate is typically 3.4-5.6 kg m^{-2}.[7]

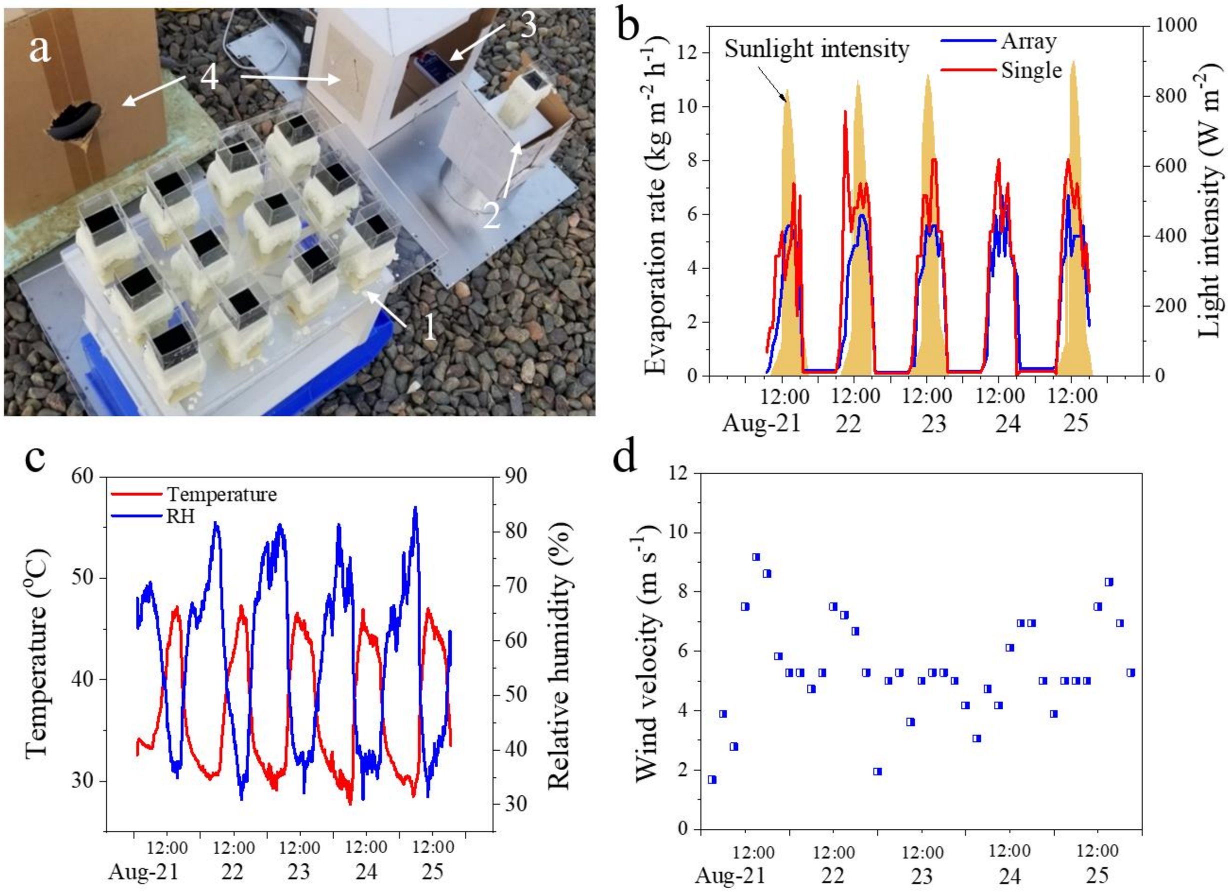

Figure S25. The experimental results of outdoor field test. (a) The photo of the outdoor field test setup. 1-solar crystallizer array, 2-single solar crystallizer as reference, 3-temperature and relative humidity sensor, 4-cameras for brine liquid level monitor. (b) The water evaporation rate of the single solar crystallizer and its array under the natural sunlight. The solar intensity measurement was missing on August 24 due to the sensor failure. (c) The temperature and relative humidity (RH) and (d) wind velocity during the field test.

Then we turned to COMSOL model to analyze the environmental thermal energy harvesting by solar crystallizer array. The model first simulates an array with the infinite repeating crystallizer units (160 mm/31 mm) to gain some theoretical insights. As presented in Figure S26a, the space among neighboring solar crystallizers is 120 mm. The cell marked by red dash line can be regarded as the duplicate cell of array

and then be selected as the research domain. The ambient temperature and relative humidity were set as 22℃ and 60%, respectively. The simulation result indicates the evaporation rate of the solar crystallizer drops from 1.67 kg m^{-2} h^{-1} to 1.36 kg m^{-2} h^{-1} after forming array when there is no incoming wind. The reduced evaporation performance of the array is slightly lower than the theoretical limit of a full solar-energy utilization (~1.5 kg m^{-2} h^{-1} under 1 sun illumination), indicating the environmental thermal energy harvesting was greatly hindered in this situation.

However, the simulation also points out that the presence of a wind field can help array harvest considerable amount of environmental thermal energy. As shown in Figure S26b, a small wind velocity of 1 m s^{-1} can help facilitate the environmental thermal energy harvesting significantly and lead to an evaporation rate of 1.65 kg m^{-2} h^{-1}. When the wind velocity further increases, more environmental thermal energy is harvested, resulting in a further enhanced water evaporation rate (Figure S26b). Such an effect can be explained by the air convection across the solar crystallizer array. Without the incoming wind, the air velocity of natural convection caused by evaporation in the simulation domain is lower than 0.1 m s^{-1}, which is restricted only within the neighboring solar crystallizers (Figure S26c). With the incoming wind, the air velocity around the crystallizers becomes much higher (Figure S26d), which in turn promotes water evaporation rate.

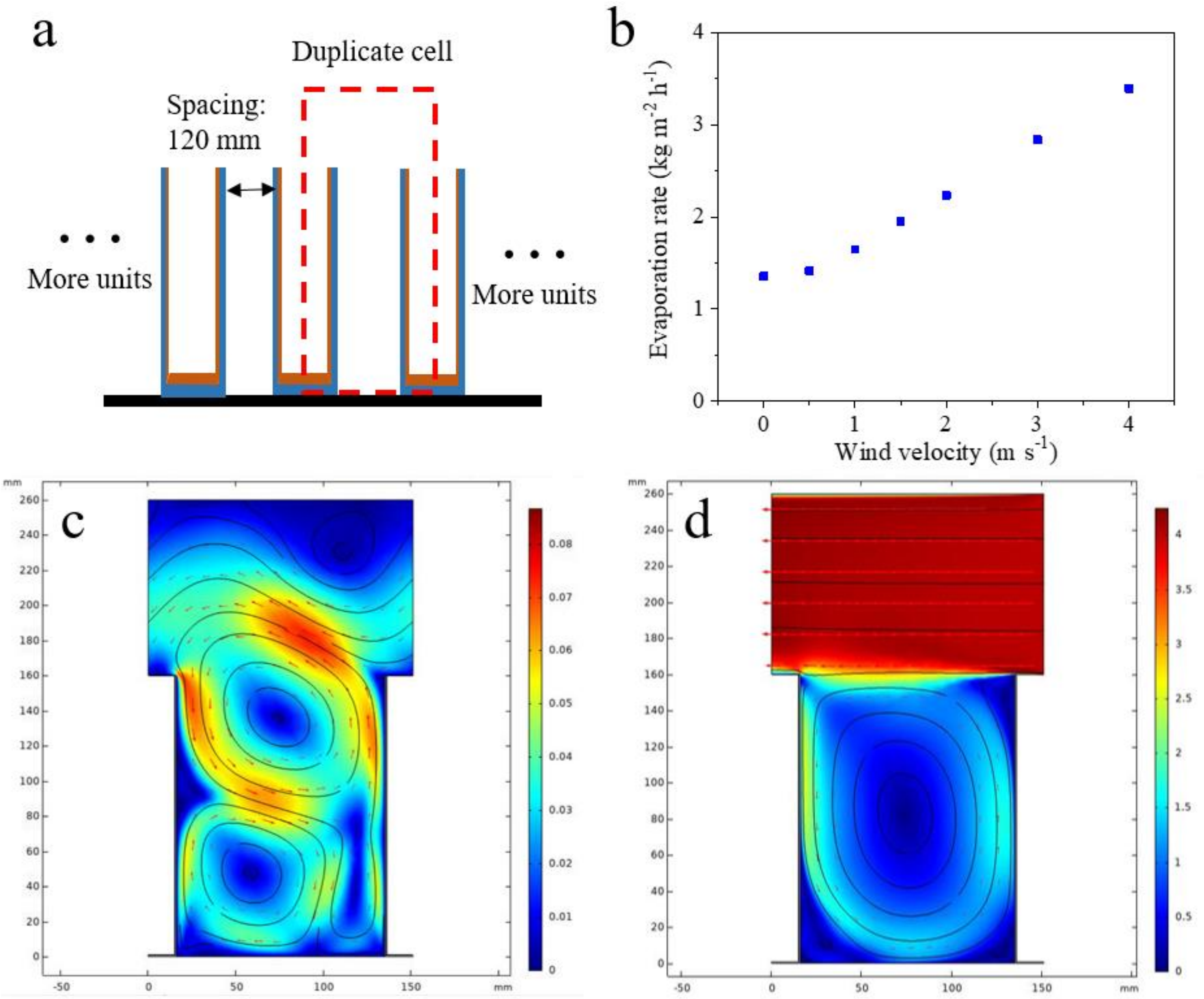

Figure S26. The configuration of the model and simulation results of the solar crystallizer array. (a) The configuration of solar crystallizer array in the simulation. (b) The performance of the simulated solar crystallizer array under incoming wind field. The simulated air velocity profiles around the solar crystallizer array (c) without wind and (d) with wind of 4 m s^{-1}.

As a summary, there are a number of important conclusions regarding scalability of the solar crystallizer design. (1) The size magnification of the solar crystallizer does not affect its performance. (2) There is indeed an inevitable small performance degradation after forming solar crystallizer array, but the overall water evaporation rate is still acceptable for practical application. (3) The presence of wind field can help solar crystallizer array gain considerable environmental thermal energy, resulting in much improved performance.

(3) The solar incident angles.

For the tilted solar incidence experiment, the solar crystallizer was placed under the solar simulator which was tilted to have desired angles. In each experiment, the distance between the top of the solar crystallizer and the light source was kept constant. The results show that the evaporation performance of the solar crystallizer with the titled solar incidence decreased to certain extent (Figure S27a) due to less solar irradiation being adsorbed. However, due to its special 3D cup structure, the solar crystallizer can still produce satisfactory water evaporation rates even when the incoming solar irradiation is not perpendicular to the base as all of the inner sides of the solar crystallizer can absorb solar irradiation (Figure S27b). Moreover, the photothermal material in this work has a high thermal conductivity, which ensures a fast thermal conduction even when the heat is generated on the top edge of the solar crystallizer.

The results of the field tests (see previous section for the details) (Figure S25b) also clearly demonstrate that the natural and daily variations of the solar incident light angel during daytime affects the evaporation but not to a decisive degree. In all, the design of the solar crystallizer can tolerate solar incident angel variation to a large extent. The solar tracking system, similar to the one used with solar PV panels, would certainly benefit the overall performance of the solar crystallizer but increase the capital cost.

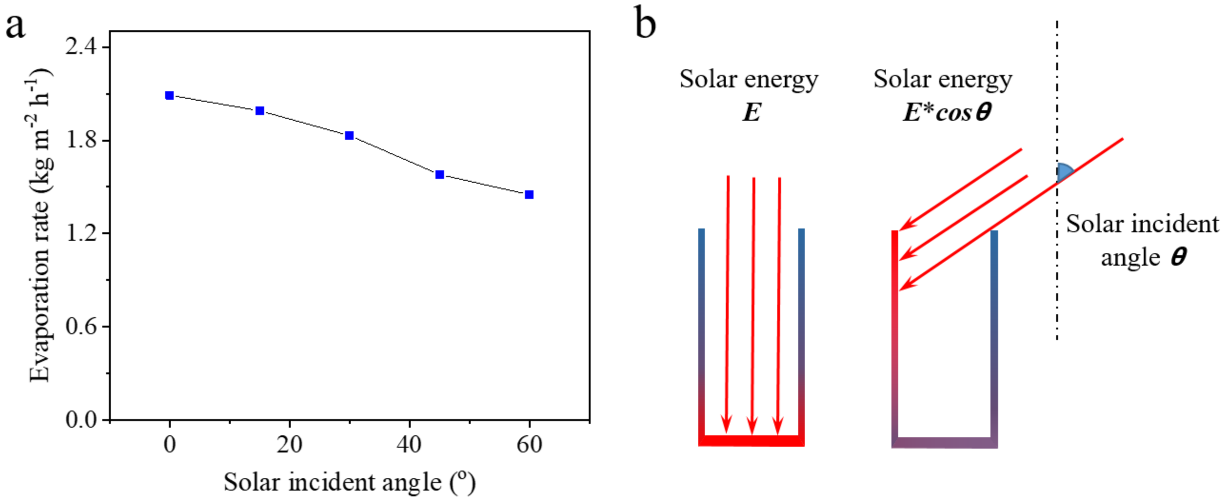

Figure S27. The influence of solar incident angle on the performance of solar crystallizer. (a) The water evaporation performance of solar crystallizer under different solar incident angle. (b) The schematic of heat transfer path inside solar crystallizer.

(4) The performance under real world condition.

The quite different water evaporation rates between indoor lab tests and outdoor field tests can be ascribed to the difference between laboratory and environmental conditions in field. COMSOL model simulation was employed to reveal the influence of ambient conditions, including ambient temperature, relative humidity and the wind field.

As presented in Figure S28, the simulation results indicate the higher ambient temperature, lower relative humidity and the presence of wind can lead to better water evaporation performance of the solar crystallizer. Based on the simulation results, the effect of wind is more significant than temperature and relative humidity.

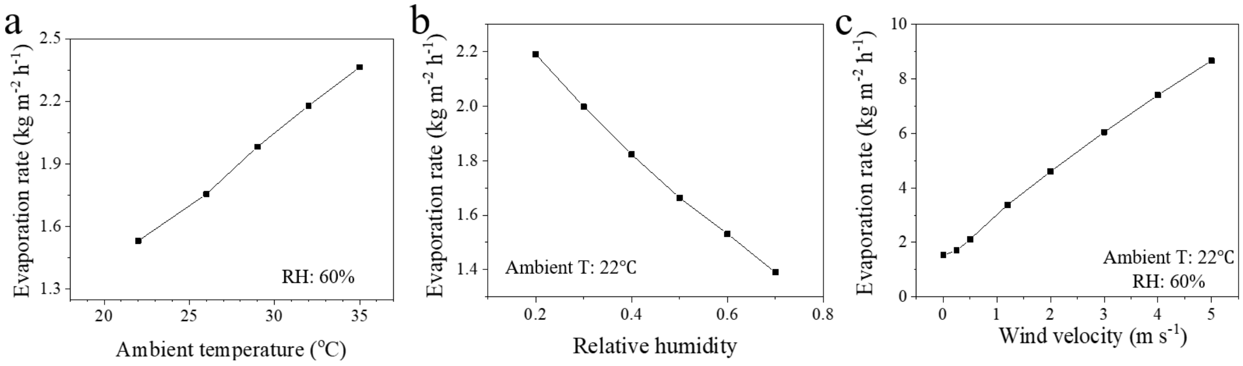

Figure S28. The COMSOL simulated water evaporation performance of the solar crystallizer (120mm/31mm) under different (a) ambient temperature, (b) relative humidity, and (c) wind velocity.

(5) Potential application scenarios.

The solar crystallizer can be a competitive alternative to the current brine crystallizer and evaporation pond for brine treatment with ZLD, especially for highly concentrated brine produced by various salt-removal processes. However, treating a sheer volume of any brine is always challenging. For instance, with 50% water recovery rate, even a small SWRO plant producing 10,000 m^3 freshwater on daily basis would have 10,000 m^3 brine with salinity of ~7 wt% to be disposed of. This is a quite large volume for any ZLD process. In this case, a hybrid design might be more attractive than a single process, with membrane-based process (e.g., RO, MD) first concentrating the brine from 7 wt% to around 20 wt% (producing 3500 m^3 of the concentrated brine) and followed by the solar crystallizer dealing with the highly concentrated brine of 20%.

We also envision that the solar crystallizer can be effectively used to treat small to medium amount of industrial brine produced by small to medium-sized industrial sectors. It is worth mentioning that under strict environmental requirement of ZLD, many small chemical plants were shut down in China and India simply due to the fact they could not afford upfront capital to procure conventional ZLD system. Given the

fact that even small commercial ZLD systems with the capacity of 5 to 100 m^3 per day need $250000 to over $2 million for equipment cost.[8] The solar crystallizer, with low barrier of entry in terms of capital investment, provides an attractive option to cost-effectively treat waste brines in the above scenarios.

References

1. Y. Shi, R. Li, Y. Jin, S. Zhuo, L. Shi, J. Chang, S. Hong, K.-C. Ng and P. Wang, *Joule*, 2018, **2**, 1171-1186.
2. C. Jia, Y. Li, Z. Yang, G. Chen, Y. Yao, F. Jiang, Y. Kuang, G. Pastel, H. Xie, B. Yang, S. Das and L. Hu, *Joule*, 2017, **1**, 588-599.
3. X. Zhou, F. Zhao, Y. Guo, Y. Zhang and G. Yu, *Energy Environ. Sci.*, 2018, **11**, 1985-1992.
4. H. S. B., *Q. J. R. Meteorol. Soc.*, 1984, **110**, 1186-1190.
5. W. M. Haynes, *CRC handbook of chemistry and physics*, CRC press, 2014.
6. C. Rodriguez-Navarro, L. Linares-Fernandez, E. Doehne and E. Sebastian, *J. Cryst. Growth*, 2002, **243**, 503-516.
7. Al-Subhi, A. M. Estimation of evaporation rates in the southern Red Sea based on the AVHRR sea surface temperature data. Journal of King Abdulaziz University: Marine Sciences, 2012, **179**, 1-26.
8. https://www.samcotech.com/how-much-will-a-zero-liquid-discharge-system-cost-your-facility/.
